# Soil-borne diseases in medicinal plants: linking allelopathy and host immunity to microbiome-based interventions

**DOI:** 10.3389/fpls.2026.1797910

**Published:** 2026-05-25

**Authors:** Wenyue Quan, Yujuan Qi, Yuan Liu, Xun Sun, Huizhen Wang, Cuiyun Zeng, Honggang Chen, Sufang Gao, Yuanyuan Xu, Qiang Zhao, Tao Du

**Affiliations:** College of Pharmacy, Gansu University of Chinese Medicine, Lanzhou, Gansu, China

**Keywords:** allelopathy, host immunometabolism, medicinal plants, microbiome engineering, pathogen virulence, soil-borne diseases

## Abstract

The rising global demand for natural medicines has intensified the cultivation of Chinese medicinal herbs, exacerbating continuous cropping obstacles and constraining the sustainable development of Chinese medicinal materials. A defining manifestation is a severe soil-borne disease that limits long-term productivity and stable medicinal quality. Despite progress in understanding individual diseases or host species, integrative syntheses of the coupled “pathogen–environment–host” network remain limited. Here, we synthesize the current understanding of soil-borne disease development under continuous cropping and evaluate ecological management options by integrating evidence on pathogen virulence, continuous-cropping-driven edaphic shifts, host immunometabolism (the coupled reprogramming of immunity and metabolism), and microbiome-based interventions. We summarize the major soil-borne pathogens and their virulence determinants and explain how allelochemical accumulation reshapes the rhizosphere microbiome, weakens the barrier functions, and facilitates pathogen ingress. We further discuss how allelochemical stress reprograms defense hormone networks (notably salicylic acid and jasmonic acid pathways) with downstream consequences for innate immune competence. Building on these insights, we assessed microbiome-guided strategies to restore rhizosphere function, including synthetic community design, recruitment of probiotic taxa, and induction of disease-suppressive soils. By integrating microbiology, chemical ecology, and plant pathology, this review provides a conceptual framework and actionable directions for the precise and sustainable control of soil-borne diseases in Chinese medicinal herb production.

## 1 Introduction

Medicinal plants are central to traditional healthcare and the modern herbal industry. Herbal medicines derived from underground organs (e.g., roots, rhizomes, bulbs, and tubers) are an important category in pharmacopeial records and are key targets for quality control and standardization (Latif and Nawaz, 2025). However, the expansion of monocultures and intensive cultivation has made soil-borne diseases, caused by pathogens that persist in soil and infect roots or the root–crown zone, a highly destructive threat to the stability and quality of medicinal production systems. Unlike staple crops, medicinal plants are valued mainly for their bioactive metabolites, such as ferulic acid in *Angelica sinensi*s (Arshad et al., 2025), ginsenosides in *Panax notoginseng* (Wei et al., 2024), and tannic acid in *Salvia miltiorrhiza* (Xu et al., 2023). The production of these compounds is strongly influenced by soil microbial communities and the local environmental conditions. Accordingly, the epidemiology of soil-borne diseases in medicinal plants often differs markedly from those in major food crops in several ways. First, many medicinal plants are grown in long-term monocultures or perennial systems, which allows the buildup of soil-borne pathogens in the rhizospheres from one season to the next. Second, root exudates and allelochemicals, bioactive secondary metabolites released by plants into the rhizosphere, can strongly shape the interactions among pathogens, beneficial microbes, and host metabolism, thereby influencing rhizosphere stability and disease development. Soil-borne pathogens reported in medicinal plants include *Fusarium* spp., *Pythium* spp., *Phytophthora* spp., *Ralstonia solanacearum*, and root-knot nematodes (*Meloidogyne* spp.). These agents can cause root rot, damping-off, blight-type diseases, bacterial wilt, and root-knot disease, respectively, and have been documented across a wide range of medicinal crops. For example, root rot is prevalent in many root- and rhizome-derived medicinal materials (e.g., *Panax* spp.) (Han et al., 2024), bacterial wilt has been reported in *Pogostemon cablin* (Sun et al., 2023b), and root-knot nematodes infect medicinal species such as *Polygonatum sibiricum* and *Peucedanum praeruptorum* (Wang et al., 2024b). Continuous cropping, nutrient imbalance, and ecological simplification disrupt the rhizosphere ecosystem, accelerating pathogen dominance and disease outbreaks (Zeeshan Ul Haq et al., 2023). This ecological imbalance simultaneously suppresses beneficial microorganisms and interferes with plant secondary metabolism, thereby compromising yield and pharmacological quality.

Over the past decade, integrated studies involving metagenomics, metabolomics, and soil ecology have indicated that outbreaks of soil-borne diseases in medicinal plants are shaped by pathogen virulence and rhizosphere dysbiosis (Xu et al., 2025). This dysbiotic state reflects an ecological imbalance in which beneficial and pathogenic interactions, nutrient cycling, and host immune signaling are disturbed. In this review, we summarize the current knowledge on the pathogenic microbiome-host continuum in medicinal plants, focusing on (i) diversity and virulence strategies of major soil-borne pathogens, (ii) roles of rotational constraints and allelochemical buildup under continuous cropping, (iii) molecular and metabolic basis of host susceptibility, and (iv) progress in microbiome-guided integrated management. We propose a system-level framework for restoring rhizosphere homeostasis and supporting sustainable production of high-quality herbal materials.

### 1.1 Literature scope and evidence synthesis

This article is an integrative narrative review. We searched the Web of Science Core Collection, PubMed, and Google Scholar for studies published between 2006 and 2026 (last searched on March 6, 2026). We used keyword combinations covering Chinese medicinal herbs and medicinal plants, soil-borne diseases, and continuous cropping (e.g., root rot, wilt, damping-off, replant disease), and rhizosphere processes (e.g., microbiome, root exudates, allelopathy, SynComs). We included peer-reviewed English articles and high-relevance Chinese studies that provided a clear host–pathogen context or reported evidence of edaphic shifts, rhizosphere dysbiosis, host immune and metabolic responses, or microbiome-based interventions. We then qualitatively synthesized the evidence within the conceptual modules presented below.

## 2 Diversity of major soil-borne pathogens

Soil-borne diseases are among the most important types of belowground diseases affecting medicinal plants and are mainly caused by soil-borne fungi, bacteria, and plant-parasitic nematodes. In contrast, most plant viruses do not fall within the narrow definition of typical soil-borne pathogens, and the diseases they cause are more appropriately classified as viral pathosystems closely associated with the soil ecosystem (García-Ordóñez and Pagán, 2024). Overall, underground diseases in medicinal plants are not always driven by a single pathogen. Instead, they often involve the successive colonization and synergistic pathogenicity of multiple pathogen groups within the same host rhizosphere or root tissues, which is also an important biological basis for disease aggravation under continuous cropping (Parrado and Quintanilla, 2024).

### 2.1 Soil-borne fungal pathogens: the main causal agents of belowground diseases in medicinal plants

Soil-borne fungal diseases are the most prevalent belowground diseases in medicinal plants and mainly include root rot, wilt, damping-off, sheath blight, southern blight, and Phytophthora blight (**Table 1**).

**Table 1:** Common soil-borne fungal diseases in medicinal plants and types of pathogens.

Disease	Symptom and infection niche	Pathogenic Organism	Infected Medicinal Plants	Continuous-cropping context	References
root rot	Root browning and decay; mainly infecting root cortex and vascular tissues	*Fusarium. Oxysporum, Fusarium. solani*	*Panax ginseng*	Perennial shade-grown monoculture; accumulation of autotoxic saponins and significant soil acidification	(Li et al., 2020a; Guan et al., 2014)
		*Fusarium oxysporum, Fusarium solani, Pythium vexans, Globisporangium spinosum, Ilyonectria mors-panacis, Cylindrocarpon destructans*	*Panax notoginseng*	Perennial shade-grown monoculture; accumulation of autotoxic saponins and significant soil acidification	(Huo et al., 2023; Ma et al., 2019; Mi et al., 2017; Mao et al., 2014; Chen et al., 2022; Lan et al., 2023)
		*Fusarium. Oxysporum*, *Fusarium solani*, *Fusarium armeniacum*, *Fusarium redolens*, *Fusarium. Commune, seudomonas marginalis, Ilyonectria mors-panacis, Pythium spinosum, Cylindrocarpon destructans*	*Pseudostellaria heterophylla*	Tuberous root monoculture; accumulation of allelopathic phenolic acids and rhizosphere dysbiosis	(Liyanapathiranage et al., 2023; Behdarvandi and Goodwin, 2023; Qi et al., 2020; Zhang et al., 2020b; Fan et al., 2021; Sun et al., 2023a; Rahman and Punja, 2005)
		*Fusarium acuminatum, Fusarium tricinctum*, *Fusarium equiseti, Fusarium solani*, *Fusarium oxysporum*, *Fusarium incarnatum, Clonostachys rosea*	*Angelica sinensis*	Deep-rooted herb monoculture; enrichment of specific allelochemicals and loss of beneficial antagonistic microbiota	(Liu et al., 2022d; Ma et al., 2022)
		*Fusarium oxysporum, Fusarium foetens*, *Fusarium avenaceum*	*Codonopsis pilosula*	Deep-rooted herb monoculture; phenolic acid accumulation and disruption of microecological balance	(Zhao et al., 2021)
		*Fusarium oxysporum, Fusarium solani, Fusarium avenaceum, Dactylonectria torresensis, Clonostachys rosea*	*Astragalus membranaceus*	Perennial deep-rooted monoculture; accumulation of specific exudates and loss of microbial diversity	(Guan et al., 2020; Qi et al., 2022; Li et al., 2021b)
		*Fusarium proliferatum, Fusarium oxysporum, Fusarium equiseti*	*Salvia miltiorrhiza*	Perennial deep-rooted monoculture; accumulation of specific exudates and loss of microbial diversity	(Yang et al., 2020; Li et al., 2022)
		*Fusarium oxysporum, Fusarium solani, Fusarium proliferatum*	*Platycodon grandiflorus*	Fleshy root monoculture; autotoxin accumulation and deterioration of the rhizosphere microenvironment	(Jeon et al., 2013)
		*Fusarium solani, Fusarium equiseti, Fusarium avenaceum*	*Atractylodes macrocephala*	Fleshy rhizome monoculture; accumulation of allelopathic lactones/volatile oils and microecological collapse	(You et al., 2013)
		*Fusarium. oxysporum, Dactylonectria torresensis, Ilyonectria coprosmae*	*Bletilla striata*	High-density bed/substrate cultivation; poor aeration and localized accumulation of secondary metabolites	(Li et al., 2020b; Duan et al., 2024; Liang et al., 2024)
		*Fusarium solani, Fusarium avenaceum, Fusarium carminascens, Fusarium oxysporum, Fusarium tricinctum, Diaporthe eres*	*Coptis chinensis*	Shade-grown perennial monoculture; accumulation of autotoxic alkaloids and prolonged rhizosphere dysbiosis	(Luo et al., 2014; Mei et al., 2020, 2021; Lyu et al., 2023)
		*Fusarium solani, Pythium ultimum*	*Paeonia officinalis*	perennial monoculture; specific root exudate toxicity and decline in beneficial root-associated microbes	(Chai et al., 2020)
		*Plectosphaerella cucumerina*	*Ligusticum chuanxiong*	Moist environment monoculture; accumulation of allelopathic lactones and microbial community shift	(Sun et al., 2024a)
Stem Rot	Brown, necrotic, and decaying lesions at the stem base; primarily infecting the crown, basal stem, and shallow roots at the soil-air interface.	*Fusarium oxysporum, Fusarium proliferatum, Ceratobasidium* sp.	*Dendrobium officinale*	High-density substrate cultivation; substrate degradation, poor aeration, and localized accumulation of root exudates	(Zhou et al., 2017)
		*Fusarium oxysporum, Fusarium foetens*	*Paris polyphylla*	Perennial shade-grown rhizome monoculture; autotoxin accumulation, and pathogen enrichment	(Zhou et al., 2018)
		*Athelia rolfsii, Sclerotium delphinii*	*Polygonatum sibiricum*	Perennial understory monoculture; soil compaction, prolonged dampness, and accumulation of fungal sclerotia at the stem base	(Tan et al., 2020; Liu et al., 2021a)
		*Fusarium. xylarioides*	*Aloe vera*	Intensive succulent plantation; soil compaction, poor drainage, and accumulation of fungal inoculum in the topsoil	(Zhu et al., 2020)
		*Fusarium. asiaticum*	*Ligusticum chuanxiong*	deep-rooted monoculture; autotoxicity and loss of microbial diversity	(Zhu et al., 2021)
Damping-off	Water-soaked constriction and rapid collapse of seedling stems (lodging); primarily infecting the hypocotyl and emerging radicles of vulnerable seedlings.	*Rhizoctonia solan, Mucor circinelloides*	*Aconitum kusnezoffii*	High-density seedling nursery monoculture; autotoxin accumulation inhibiting early root development and enriching soil-borne inocula	(Cui et al., 2020)
		*Rhizoctonia solani*	*Lancea*	Continuous nursery bed cultivation; accumulation of specific allelochemicals weakening seedlings and massive enrichment of fungal sclerotia	(Hassan and Chang, 2020, 2021)
southern blight	Fan-like white mycelial mats and mustard-seed-like brown sclerotia forming on the host surface; strictly targeting the crown and upper root zone.	*Sclerotium rolfsii Sacc.*	*Aconitum carmichaelii*; *Anoectochilus; Dendrobium brownie*; *viridulum*	Mixed high-density or substrate cultivation; massive accumulation of highly resistant sclerotia in soil debris	(Wang et al., 2018; Chen et al., 2022) (Wu et al., 2023) (Yang et al., 2007)
		*Sclerotium rolfsii Sacc, Atheliarolfsii*	*Coptis chinensis*	Perennial shade-grown monoculture; dense canopy humidity facilitating explosive mycelial spread	(Tang et al., 2024)
		*Athelia rolfsii*	*Pogostemon cablin*	High-density warm-humid monoculture; allelochemical accumulation weakening basal stems alongside exponential growth of soil-borne sclerotia	(Lu et al., 2025) (Wen et al., 2024)
Sclerotinia disease	Water-soaked soft rot with dense, cottony white mycelium and irregular large black sclerotia; infecting the crown, stems, and sometimes foliage under high humidity.	*Sclerotium sclerotiorum*	*Panax quinquefolius*; *Artemisia capillaris; Cannabis sativa*; *Pinellia ternata*	High-density or shade-grown continuous cropping; dense, humid canopies favoring explosive outbreaks and accumulation of highly resistant sclerotia in soil debris	(Guan et al., 2022; Liu et al., 2023b; Wang et al., 2020) (Garfinkel, 2021)
Wilt	Systemic leaf yellowing, drooping, and distinct vascular discoloration (browning); pathogens penetrate the root cortex and systemically colonize the xylem vessels.	*Verticillium longisporum*	Medicinal Plants of Brassicaceae	Deep-rooted herb monoculture; persistent accumulation of highly resistant microsclerotia in the soil profile and allelopathy-induced root vulnerability	(Depotter et al., 2016)
			*Scutellaria baicalensis*		(Dung et al., 2011)

Overall, fungal belowground diseases in medicinal plants are dominated by *Fusarium* spp., often with co-involvement of *Pythium* spp., *Rhizoctonia solani*, and other soil-borne fungi. Root rot is the most common syndrome, whereas standardized information on infection niche, continuous-cropping duration, and pathogen-complex structure remains limited in many medicinal-plant systems.

Among these diseases, root rot is the most common and destructive, typically causing browning, necrosis, and decay of roots and tuberous roots (Bischoff Nunes and Goodwin, 2022). Current studies indicate that fungal diseases in medicinal plants are often centered on *Fusarium* spp., with *Pythium* spp. and *Rhizoctonia solani* frequently involved in co-infection (Li et al., 2024a; Guan et al., 2014). Many medicinal plants are harvested for their roots or rhizomes, have long cultivation cycles, and contain nutrient-rich underground tissues, making them particularly susceptible to infection by soil-borne fungi.

### 2.2 Soil-borne bacterial diseases: reduced diversity but severe impairment of medicinal material quality

Compared to fungal diseases, soil-borne bacterial diseases of medicinal plants are less well documented; however, they often cause more direct damage to the appearance, storage stability, and processability of root and rhizome medicinal materials (**Table 2**). The major diseases include bacterial wilt, soft rot, and certain root rots, which typically present with tissue water-soaking or dehydration, rapid cell wall degradation, vascular browning, and root/rhizome softening and decay. For example, soft rot pathogens such as *Pectobacterium* rapidly destroy host tissues via cell wall-degrading enzymes, causing maceration, tissue collapse, and wet rot disintegration (Kvitko et al., 2025).

**Table 2:** Common soil-borne bacterial diseases in medicinal plants and pathogen types.

Disease	Symptom and infection niche	Pathogenic organism	Infected medicinal plants	Continuous-cropping context	References
Bacterial Root Rot	Water-soaked, foul-smelling soft decay of root tissues; pathogens primarily invade through mechanical/nematode wounds or natural openings, rapidly colonizing and degrading the cortical parenchyma and vascular bundles.	*Pseudomonas aeruginosa*	*Panax ginseng*	Perennial shade-grown monoculture; autotoxic saponin accumulation compromises root epidermal integrity, creating micro-wounds that facilitate bacterial chemotaxis and invasion.	(Gao et al., 2014; Guo et al., 2022)
Bacterial Wilt	Acute wilting of foliage while remaining green; vascular bundles turn brown and exude milky bacterial ooze when severed. Pathogens enter via root wounds and systemically colonize the xylem.	*Ralstonia pseudosolanacearum*	*Zingiber officinale*	Continuous rhizome cultivation; accumulation of pathogenic Ralstonia in soil and latent infections in seed rhizomes, often exacerbated by high soil temperature and moisture.	(Soto-Heredia et al., 2024; Chiu et al., 2025)
Soft Rot	Rapid maceration, water-soaking, and foul-smelling decay of fleshy organs. Pathogens secrete cell wall-degrading enzymes (pectinases), primarily infecting parenchymatous tissues via micro-wounds or lenticels.	*Pectobacterium aroidearum, Pectobacterium carotovorum*	*Pinellia ternata*	High-density tuber monoculture; specific root exudates alter the rhizosphere microbiome assembly and severely suppress host physiological defenses, facilitating massive invasion by Pectobacterium spp.	(Du et al., 2023; Wang et al., 2021; Ying et al., 2007)
		*Lelliottia. aquatilis*	*Codonopsis pilosula*	Fleshy root monoculture; continuous cropping drives micro-ecological degradation and increases soil-borne pest damage, providing vital entry wounds for soft rot pathogens.	(Zhao et al., 2022b)
		*Pectobacterium brasiliense*	*Aconitum carmichaelii*	High-density tuberous root cultivation; warm and waterlogged soil conditions combined with monoculture lead to exponential enrichment of Pectobacterium in the topsoil.	(Zhang et al., 2022)
Crown Gall	Formation of tumor-like galls on the roots and crown (root-shoot junction). Agrobacterium transfers T-DNA into the plant genome, inducing uncontrolled cell division via wound sites.	*Agrobacteriumtumefaciens*	*Angelica sinensis*	Deep-rooted herb monoculture; increased nematode and insect damage during continuous cropping creates abundant entry wounds, allowing persistent soil-borne Agrobacterium to initiate infection.	(Zhang, 2023)

Compared with fungal diseases, documented bacterial soil-borne diseases are fewer in number but often cause more direct deterioration in the appearance, storage stability, and processability of root- and rhizome-derived medicinal materials. Current evidence remains uneven across host species, and more systematic data are still needed on infection routes, host predisposition, and interactions with nematode injury or rhizosphere imbalance.

Recent studies have provided direct evidence of bacterial involvement in underground diseases of medicinal plants, including *Panax notoginseng* (Zou et al., 2023). Broad-host-range soil-borne bacterial pathogens, such as *Ralstonia solanacearum*, indicate that medicinal plants face an elevated infection risk under root injury, nematode damage, or rhizosphere imbalance (Senuma et al., 2025; Wang et al., 2025).

### 2.3 Plant-parasitic nematodes: direct pathogens and key predisposing factors of complex diseases

Plant-parasitic nematodes infect roots, fibrous roots, and underground stems, causing root galls, necrosis, deformity, and impaired nutrient uptake, which directly reduce the yield and quality of medicinal plants (Yang et al., 2023a; Zhou et al., 2024; Ni et al., 2023). More critically, nematodes play a dual role in underground diseases: they directly damage root tissues, while creating favorable conditions for secondary fungal and bacterial infections via wounding, altered nutrient allocation, and modified local microenvironments. Current studies have revealed marked differences in the susceptibility of medicinal plants to key parasitic nematodes (e.g., root-knot nematodes), with damage frequently coinciding with continuous cropping obstacles. *Panax notoginseng* exhibits age-dependent resistance, with seedlings being highly susceptible and mature plants exhibiting adult-plant resistance (Wang et al., 2023). Dominant root-knot nematode species also vary across hosts such as *Polygonatum sibiricum*, *Peucedanum praeruptorum*, and *Aconitum carmichaelii* (Zhou et al., 2024). Mechanistically, nematode-induced wounding and nutrient reallocation promote infection by secondary pathogens, including *Fusarium*, *Pythium*, and soft rot bacteria (Parrado and Quintanilla, 2024). Thus, plant-parasitic nematodes act not only as independent pathogens but also as critical mediators connecting root injury, rhizosphere dysbiosis, and multi-pathogen complex infections. The Common nematode diseases affecting medicinal plants are summarized in **Table 3**.

**Table 3:** Species of medicinal plants infected by common nematode diseases.

Disease	Symptom and infection niche	Pathogenic organism	Infected medicinal plants	Continuous-cropping context	References
Root-knot Nematode Disease	Formation of distinct galls (knots) on the root system, causing severe stunting and yellowing of above-ground parts. Infective juveniles penetrate root tips, establish specialized feeding sites (giant cells) in the vascular cylinder, and disrupt nutrient/water transport.	*Meloidogyne hapla*	*Salvia miltiorrhiza*; *Aucklandia costus*; *Atractylodes macrocephala*; *Bupleurum*; *Siegesbeckia orientalis*; *Panax notoginseng*	Perennial/biennial root herb monoculture in temperate regions; continuous cropping disrupts antagonistic soil microfauna, allowing explosive population growth of overwintering juveniles.	(Pan et al., 2022) (Wu et al., 2021a) (Huang et al., 2022) (Shi et al., 2022) (Yang et al., 2024) (Dong et al., 2013)
		*Meloidogyne javanica*	*Zingiber officinale*; *Bletilla striata*	Rhizome/tuber continuous cropping in warm climates; accumulation of specific root exudates acts as strong chemoattractants for nematodes, coupled with the buildup of egg masses in unharvested debris.	(Hajihassani et al., 2019) (Yang et al., 2023b)
		*Meloidogyne arenaria*	*Ophiopogon japonicus*; *Cynanchum atratum*; *Atractylodes macrocephala; Morinda officinalis*	High-density fleshy root/rhizome monoculture; degradation of the soil physical structure and rhizosphere immunity facilitates multi-generational overlapping and rapid nematode proliferation	(Wu et al., 2021b) (Qin et al., 2022a) (Wang et al., 2012) (Fu et al., 2013)
		*Meloidogyne enterolobii*	*Solanum nigrum*	Long-term cultivation; continuous host presence drives selection pressure, favoring the dominance of highly aggressive and multi-host compatible nematode species.	(Chen et al., 2023a)
		*Meloidogyne incognita*	*Dioscorea opposita*; *Salvia miltiorrhiza*	Deep-rooted fleshy tuber/root monoculture; repeated monoculture eliminates natural nematode predators and leads to critical accumulation of infectious second-stage juveniles (J2) in the rhizosphere.	(Gao et al., 2023) (Wen et al., 2022)

Plant-parasitic nematodes function not only as direct pathogens but also as key predisposing factors in complex belowground disease systems by creating wounds and modifying local nutrient and microbial environments. Across medicinal plants, susceptibility, dominant nematode species, and their interactions with secondary fungal or bacterial infection remain strongly host- and context-dependent.

### 2.4 Soil-associated viral pathogens: vector-dependent transmission

Most plant viruses are primarily transmitted by living vectors and are not strictly classified as soil-borne diseases. In this review, we define these diseases as soil-associated viral disease systems, where soil acts mainly as a temporary reservoir for virus particles, virus-bearing plant residues, or transmission vectors rather than as the primary transmission route. Only a few highly environmentally stable viruses can retain infectivity in soil for a short period and enter the host via root micro-wounds (**Table 4**).

**Table 4:** Transmission mechanisms and evidence-based management of soil-associated viruses in medicinal plants.

Virus classification and representative viruses	Primary medicinal hosts and continuous-cropping context	Transmission routes and vectors	Strength of evidence	Management priority	References
*Tobamovirus* (Rehmannia mosaic virus, ReMV)	*Rehmannia glutinosa* (asexual propagation via underground tuberous roots, high-frequency continuous cropping)	Mechanical transmission via contact with diseased residues and root micro-wounds	High (host and pathogenicity confirmed; transmission route fully validated by experiments)	Virus-free seedlings; diseased residue removal	(Komiyama et al., 2021; Qin et al., 2022b; Koh et al., 2017)
*Potexvirus* (Cymbidium mosaic virus, CymMV)	*Dendrobium officinale, Bletilla striata* (substrate continuous cropping, high-density bed cultivation system)	Mechanical transmission via contact with virus-carrying residues in soil/substrate and agricultural operations	Moderate (host confirmed; contact/mechanical transmission well-supported by genus-level evidence)	Regular monitoring; clean seedlings	(Yusop et al., 2022; Pai et al., 2020)
*Nepovirus* (Arabis mosaic virus, ArMV, etc.)	*Mentha* spp. (perennial or high-density continuous cropping system)	Transmission via root feeding by nematode vectors Xiphinema spp. or Longidorus spp.	Moderate (strong epidemiological association; host-specific transmission in medicinal plants unconfirmed)	Nematode control; weed management	(Tzanetakis et al., 2010; Harrison and Cadman, 1959; Valdez et al., 1974)
*Tobravirus* (Tobacco rattle virus, TRV)	*Paeonia lactiflora* (medicinal parts are underground roots, perennial continuous cropping)	Transmission via piercing and sucking on root epidermis by stubby-root nematodes Trichodorus spp.	Moderate–High (vector transmission mechanism well-established in model systems; direct evidence in medicinal host system is limited)	Nematode control; early monitoring	(Zhen and Chen, 2016; Vlasava et al., 2024; Hernández et al., 1997)
*Alphanecrovirus* (Tobacco necrosis virus A, TNV-A)	*Cannabis sativa* (continuous cropping environment with artificial shading and high soil moisture)	Fungus-vectored transmission via zoospores of Olpidium brassicae during root infection	Moderate–High (fungal vector mechanism well-validated; direct evidence in Cannabis sativa pathosystem is limited)	Water source control; targeted intervention for vector fungi	(Lopez-Jimenez et al., 2025; Teakle, 1960, 1962)

Soil-associated viral disease systems here refer to cases in which soil acts mainly as a temporary reservoir for virus particles, infected residues, or vectors rather than as the primary transmission route. Strength of evidence refers to support for the specific medicinal host–virus–transmission combination: High, directly validated in the same or a closely comparable system; Moderate–High, mechanism well established in related systems but incompletely validated in the medicinal host system; Moderate, host confirmed with strong epidemiological association but limited host-specific transmission evidence. Management priority reflects the main transmission link requiring control in each system.

Overall, living vectors, such as nematodes and fungi, remain the key link in the transmission of these viruses. Therefore, the focus of control is not solely on soil treatment but also on breaking the transmission chain and managing vector populations. Existing evidence has confirmed soil-associated viral diseases in medicinal plants, including *Rehmannia glutinosa* and Panax notoginseng (Chen et al., 2014). However, current studies mostly focus on pathogen identification and mixed infections, with limited direct evidence for specific transmission routes. Further research is required to clarify the transmission modes and management priorities of these viruses.

## 3 Pathogen complexes and synergistic infection

In continuous cropping systems of medicinal plants, the development of soil-borne diseases is increasingly recognized as a systemic process driven by pathogen complexes rather than the isolated action of individual pathogens (Parrado and Quintanilla, 2024). The concept of pathogen complexes encompasses not only the co-occurrence of diverse pathogens (including fungi, bacteria, and nematodes) in the same host or rhizosphere niche but also their ability to form functional synergies. These synergies arise through sequential infection, tissue damage, nutrient complementation, host immune modulation, and rhizosphere microecosystem remodeling, ultimately resulting in significantly higher disease severity than single-pathogen infections (Chen et al., 2026). In medicinal plants, root rot of Panax notoginseng is no longer simply regarded as a single Fusarium-related disease, but is now considered a multi-pathogen system involving Fusarium solani, F. oxysporum, Ilyonectria mors-panacis, Plectosphaerella plurivora, Pythium vexans, and certain bacterial members (Li et al., 2024b). Similarly, root rot of *Angelica sinensis* has also been reported to be closely associated with the *Fusarium* species complex and may occur together with other fungal members (Cheng et al., 2025), indicating that such a complex pattern is not uncommon in major root medicinal crops.

The core priority of complex infection research is not merely the detection of co-occurring pathogens but the identification of which pathogens are consistently involved in disease progression, the dynamics of their load, and whether they act synergistically within specific temporal windows and tissue niches. Thus, a standardized workflow spanning broad-spectrum discovery, targeted validation, dynamic quantification, and spatial tissue localization is essential for the robust characterization of pathogen complexes (Whale et al., 2020). High-throughput sequencing is suitable for screening candidate complex members and analyzing community shifts, with its core strengths lying in novel pathogen discovery and correlation analysis (Massart et al., 2022). In contrast, qPCR and dPCR are more appropriate for the quantitative validation of key members, especially for comparing the load dynamics of fungi, bacteria, and nematodes across different disease stages and ecological niches (Stephen et al., 2009). For soil-borne diseases of medicinal plants, such detection should cover the asymptomatic, early onset, and typical symptomatic stages as much as possible, with simultaneous sampling of root tissue, rhizosphere soil, and bulk soil (Ho et al., 2024). This design avoids misidentifying secondary colonizers or opportunistic saprophytes as core pathogenic members of the pathogen complex (Darriaut et al., 2024). Drawing on studies of root rot disease complexes in legumes and related plant pathosystems, researchers integrate pathogen detection with systematic analyses of the temporal dynamics, spatial distribution, and abundance shifts of target microbes to avoid misclassifying microbes that are only enriched post-disease onset as core components of the pathogenic complex (Yilma et al., 2025).

The key to research on complex pathogens lies not in identifying the coexistence of multiple pathogens but in verifying whether they act synergistically to promote disease (Parrado and Quintanilla, 2024). Therefore, the standard of evidence should be higher than that for single pathogen identification, requiring the establishment of a complete evidence chain through combined or sequential inoculation, interactive statistics, re-isolation and inoculation tests, and mechanistic validation. Based on the mechanism of complex infection, the control of soil-borne diseases in medicinal plants should not be limited to suppressing a single pathogen but should shift to the comprehensive regulation of the entire pathogenic complex and rhizosphere microecology (Deng et al., 2024). Priority should be given to identifying pioneer pathogens, amplifying pathogens, and secondary colonizers, and implementing hierarchical management according to the disease stage. Meanwhile, measures such as crop rotation, soil amendment, optimized organic inputs, and functional microbial community reconstruction should be integrated to restore soil suppressiveness (Guo et al., 2022; Parrado and Quintanilla, 2024). In the future, efforts should be strengthened to integrate multi-pathogen joint detection, dynamic monitoring, time-series verification, and rhizosphere ecological management. The mechanism of disease development should be understood at the level of pathogenic complexes, with simultaneous attention to changes in the yield and quality of medicinal plants.

## 4 Virulence mechanisms

### 4.1 Host recognition and mechanisms of infection

Within the highly complex soil–rhizosphere environment of medicinal plants, soil-borne pathogens depend on chemical signaling to shift from a free-living state in the soil to active invasion of host roots. Root exudates include primary metabolites such as low-molecular-weight organic acids (e.g., malic acid, citric acid, and fumaric acid), sugars (e.g., glucose and sucrose), and amino acids, as well as secondary metabolites including phenolics/phenolic acids (e.g., ferulic acid) and flavonoids (Halshoy et al., 2025). Some of these secondary metabolites act as allelochemicals that modulate rhizosphere microbial assembly during continuous cropping (Anderson, 2023). Root exudates establish stable concentration gradients in the rhizosphere, which are detected by methyl-accepting chemotaxis proteins on the pathogen surface, thereby driving flagellum-dependent chemotactic movement toward roots. Pathogen chemotaxis is not simply a response to nutrient availability; it reflects the targeted perception of host-derived signals and represents an integral component of virulence regulation in pathogens (Matilla and Krell, 2018). Root exudates attract pathogens and guide hyphal tips or motile propagules along concentration gradients toward susceptible root apices, thereby initiating infection (Leroy et al., 2021). For instance, ginseng root exudates attract soft rot bacteria and facilitate their colonization, demonstrating that chemotaxis-mediated recognition of medicinal plant exudates by pathogenic bacteria is a critical early step in disease establishment (Lei et al., 2017). In addition to serving as a carbon source, medicinal-plant root exudates are instrumental in shaping rhizosphere community assembly by selectively recruiting specific microbial groups (Massalha et al., 2017). In this context, root exudation can function as a distress signaling strategy to attract beneficial microorganisms. Notably, organic acids released by *P. notoginseng* roots, particularly fumaric acid, promote the recruitment and proliferation of beneficial bacteria, thereby suppressing root rot pathogens (Ma et al., 2021). However, chemotaxis alone is insufficient for successful infection. Following chemotactic accumulation at the rhizoplane, pathogens commonly activate quorum-sensing systems to coordinate their collective behavior during host invasion.

Soil-borne pathogens typically enter plants through the root system, where they initially colonize the cortex in a largely asymptomatic manner before accessing the xylem and systemically spreading through vascular tissues. Using live cell imaging, Tian et al (Tian and Kong, 2022). clearly visualized the successive stages of *V. dahliae* infection, including attachment to the root epidermis, penetration of cortical tissues, and extensive proliferation within the vascular bundles. Some pathogens form specialized infective structures on the root surface and rapidly disrupt epidermal and cortical cells through the intense secretion of cell wall-degrading enzymes (CWDEs) and toxins. Many soil-borne pathogens adopt intermediate infection strategies that combine localized cortical necrosis with subsequent colonization of xylem vessels to achieve effective systemic invasion.

### 4.2 Virulence factors

#### 4.2.1 Effector proteins

During the infection of medicinal plants, pathogen virulence is largely mediated by CWDEs, effector proteins, and toxins. Proteins containing LysM domains are particularly widespread among fungal effectors. A well-studied example is *Cladosporium fulvum*, the causal agent of tomato leaf mold, which produces the core LysM effector Ecp6 (De Jonge et al., 2010). This protein sequesters chitin oligosaccharides in the apoplast, thereby preventing receptor dimerization, blocking the host’s perception of fungal invasion, and ultimately suppressing immune activation. Similarly, LysM effectors from *V. dahliae* bind to chitin fragments, attenuate chitin-induced immune responses, and protect fungal hyphae from plant hydrolytic enzymes (Kombrink et al., 2017). Collectively, LysM-containing effectors act by tightly binding to chitin released from fungal cell walls and inhibiting chitin-mediated pattern-triggered immunity.

In addition to apoplastic effectors, some fungal effectors are translocated into the host nucleus, where they interfere with defense-related transcription factors (TFs) or chromatin-associated processes, thereby suppressing effector-triggered and basal immunity at downstream regulatory levels. For instance, the *V. dahliae* effector Vd424Y localizes to the host nucleus and modulates effector-triggered immune responses (Liu et al., 2021b). Another rapidly expanding area of research concerns cross-kingdom small RNA effectors. *Botrytis cinerea* can deliver small RNAs into plant cells via extracellular vesicles (Weiberg et al., 2013). These small RNAs associate with host Argonaute complexes and silence defense-related genes, further compromising plant immunity.

Soil-borne bacterial Type III secretion system effectors deliver proteins into the root cortex or xylem parenchyma cells, suppressing pattern-triggered immunity (PTI) and effector-triggered immunity (ETI), while modulating salicylic acid (SA), jasmonic acid (JA), and ethylene (ET) signaling networks. PTI is the first layer of receptor-mediated basal defense in plants, whereas ETI is a stronger intracellular defense response, typically mediated by NLR proteins. For instance, *R. pseudosolanacearum*, the causal agent of ginger bacterial blight, encodes RipBJ and RipBO (Suraby et al., 2022), and the Type III secretion system effector AvrPto of *P. syringae* binds to receptor kinases FLS2 and EFR to block PTI (Xiang et al., 2008). Although bacterial and fungal effectors differ structurally and mechanistically, they functionally converge by targeting pathogen-associated molecular pattern (PAMP) recognition, hormonal pathways, and transcriptional regulation. PAMP recognition is the core initiating step in plant innate immunity. This refers to the biological process by which plants perceive evolutionarily conserved microbial signature molecules via cell surface receptors, which is a prerequisite for triggering PTI.

#### 4.2.2 CWDEs and toxins

Several soil-borne fungi produce toxins that are classified as mycotoxins or resorcylic acid lactones (RALs) (Kuttikrishnan et al., 2021). In ginseng root rot, highly virulent strains of the dominant pathogen *I. mors-panacis* produce multiple RALs, whereas low-virulence or non-pathogenic strains produce few or none. Metabolomic analyses have indicated that these RALs accumulate prominently in diseased roots and are closely associated with root browning, tissue disintegration, and the “root rot disappearance” phenotype (Jacob et al., 2022). CWDEs also accumulate extensively at infection sites across various plants (Wibberg et al., 2016), thereby synergistically hydrolyzing pectin, hemicellulose, and structural proteins to rapidly soften and necrotize the epidermis and cortex. This process is particularly detrimental to medicinal plants with enlarged roots, such as ginseng and angelica. Both CWDEs and toxins disrupt plant exosome functions, thereby compromising host immunity.

Fungi produce substantial amounts of diverse CWDEs, primarily from the glycoside hydrolase family (Rawat et al., 2019). For instance, during ginseng infection, *Infusoria morpanacis* secretes large quantities of hydrolases, such as cellulases and pectinases, facilitating rapid penetration of the root epidermis and subsequent spread through the cortex and internal tissues (Farh et al., 2018). Certain bacteria also release extracellular degradative enzymes, including proteases and cellulases, via type II secretion systems to disrupt plant tissue (Amna et al., 2019). CWDE activity further generates damage-associated molecular patterns, such as oligogalacturonides, which are recognized by plant-specific receptors to trigger PTI (Pontiggia et al., 2020; Pastor et al., 2022). Therefore, the effective regulation of CWDEs requires balancing their role in tissue penetration to avoid the excessive activation of plant immune responses.

#### 4.2.3 Virulence-regulatory network

Pathogenic factors do not function in isolation but are coordinated within integrated virulence regulatory networks. Quorum sensing is the most prevalent regulatory system that controls the virulence of soil-borne bacteria. When signaling molecules accumulate to a critical threshold, quorum sensing activates coordinated gene expression, synchronizing collective behaviors, such as rhizosphere colonization and virulence factor production, thereby enabling successful root infection. For instance, the phc quorum-sensing system in *Ralstonia solanacearum* regulates extracellular polysaccharide production, biofilm formation, and extracellular enzyme secretion, thereby facilitating effective rhizosphere colonization and vascular invasion. Quorum sensing disruption by specific inhibitors markedly reduces bacterial virulence (Yoshihara et al., 2020). The RasI/R system within the quorum-sensing network of *Penicillium chrysogenum* senses bacterial population density through *AHL* signaling and regulates cellulase production, motility, biofilm formation, oxidative stress response, and virulence in *R. solanacearum* EP1. Disruption of RasI/R markedly reduces bacterial wilt symptoms in host plants, such as tomatoes (Yan et al., 2022). In soft rot bacteria associated with medicinal plants, the core gene responsible for quorum sensing, which encodes the AHL synthase ExpI, controls the production of plant CWDEs and the severity of soft rot symptoms and influences bacterial colonization and transmission (Kang et al., 2024).

Regulating virulence of soil-borne fungi is not confined to a single pathway. The Velvet complex, a conserved fungal regulatory complex linking development and secondary metabolism, including VeA, VelB, and LaeA, governs hyphal and spore development, toxin biosynthesis, and secondary metabolism, thereby providing a physiological basis for root colonization and tissue necrosis (Sun et al., 2024b; Huang et al., 2025). In parallel, mitogen-activated protein kinase (MAPK) cascades and autophagy-associated phosphatases (Liu et al., 2023a), which act as negative regulators, function as upstream signaling frameworks that modulate the transcription of effector genes, toxins, and CWDEs. Collectively, the virulence of soil-borne pathogenic fungi is governed by interconnected signaling and transcriptional networks. Representative modules include MAPK-related pathways and the Velvet complex, which together form an integrated regulatory system controlling infection-associated traits (Li et al., 2024d; Cano et al., 2022).

## 5 Soil ecological imbalance during the development of continuous-cropping obstacles

In medicinal plant production systems, continuous cropping obstacles are aligned with what is traditionally termed soil degradation, replant disease, or continuous cropping syndrome. In essence, these obstacles are not driven by a single pathogenic factor but arise from the combined effects of multiple processes under long-term monoculture, including shifts in soil physicochemical properties, allelochemical accumulation, and rhizosphere microbial community imbalance. With increasing years of continuous monocropping, medicinal plants typically display declining yields, exacerbated disease incidence and severity, and deteriorating medicinal quality. During this process, allelochemicals released from root exudates and decomposing plant residues accumulate progressively and exert autotoxic effects on host plants. These chemical signals interact with changes in soil nutrient status, pH, and microbial community structure, further driving pathogen enrichment and impairing the functional activity of beneficial microorganisms. This cascade of effects ultimately triggers the onset of soil-borne diseases and exacerbates continuous cropping obstacles (Goodwin, 2022; Liu et al., 2022c; Liao and Xia, 2024).

Under continuous cultivation of medicinal plants, disease outbreaks often occur abruptly and become difficult to control once the pathogen population reaches a critical threshold. Studies on soils continuously cropped with ginseng and *Rehmannia* have shown a marked increase in the relative abundance of pathogenic fungi and oomycetes, including *Fusarium*, *Ilyonectria*, *Cylindrocarpon*, *Rhizoctonia*, and *Phytophthora*. In parallel, beneficial microorganisms have significantly declined, including plant growth-promoting rhizobacteria, arbuscular mycorrhizal fungi, and antagonistic taxa such as *Trichoderma hamatum* (Goodwin, 2022; Liu et al., 2022c). These patterns indicate that continuous cropping disrupts the balance between pathogenic and beneficial microorganisms, ultimately leading to dysbiosis of the rhizosphere microbiome and the microbial community at the root–soil interface.

Soils under continuous cultivation of medicinal plants tend to accumulate high levels of allelochemicals that are structurally similar to the bioactive compounds in these plants. Representative examples include saponins from ginseng and *P. notoginseng*, phenolic acids from *S. miltiorrhiza*, and alkaloids from *Sophora* spp. Many phenolic acids and terpenoids exert pronounced autotoxic effects on conspecific plants at field-relevant concentrations, including the inhibition of root elongation, disruption of membrane integrity, and induction of oxidative stress (Wang et al., 2024a). Some allelochemicals can stimulate spore germination, hyphal growth, chemotaxis, and expression of virulence genes in soil-borne pathogens, whereas others favor the competitive establishment of beneficial microorganisms (Ma et al., 2021). In addition, specific flavonoids or phenolic acids have been identified as pathogenic chemotactic signals, such as *Fusarium* and *Rhizoctonia*, which markedly increase their accumulation in the rhizosphere and the likelihood of infection (He et al., 2022a). The functional boundary between these compounds, acting as medicinal constituents or autotoxic allelochemicals, largely depends on their concentration, soil-binding state, and microbial transformation process (Li et al., 2020c; Wang et al., 2023a; Rabas et al., 2025). Collectively, obstacles to continuous cropping emerge from long-term interactions among plants, soil physicochemical properties, and soil microbial communities.

### 5.1 Types and accumulation of allelochemicals

**Figure 1:**
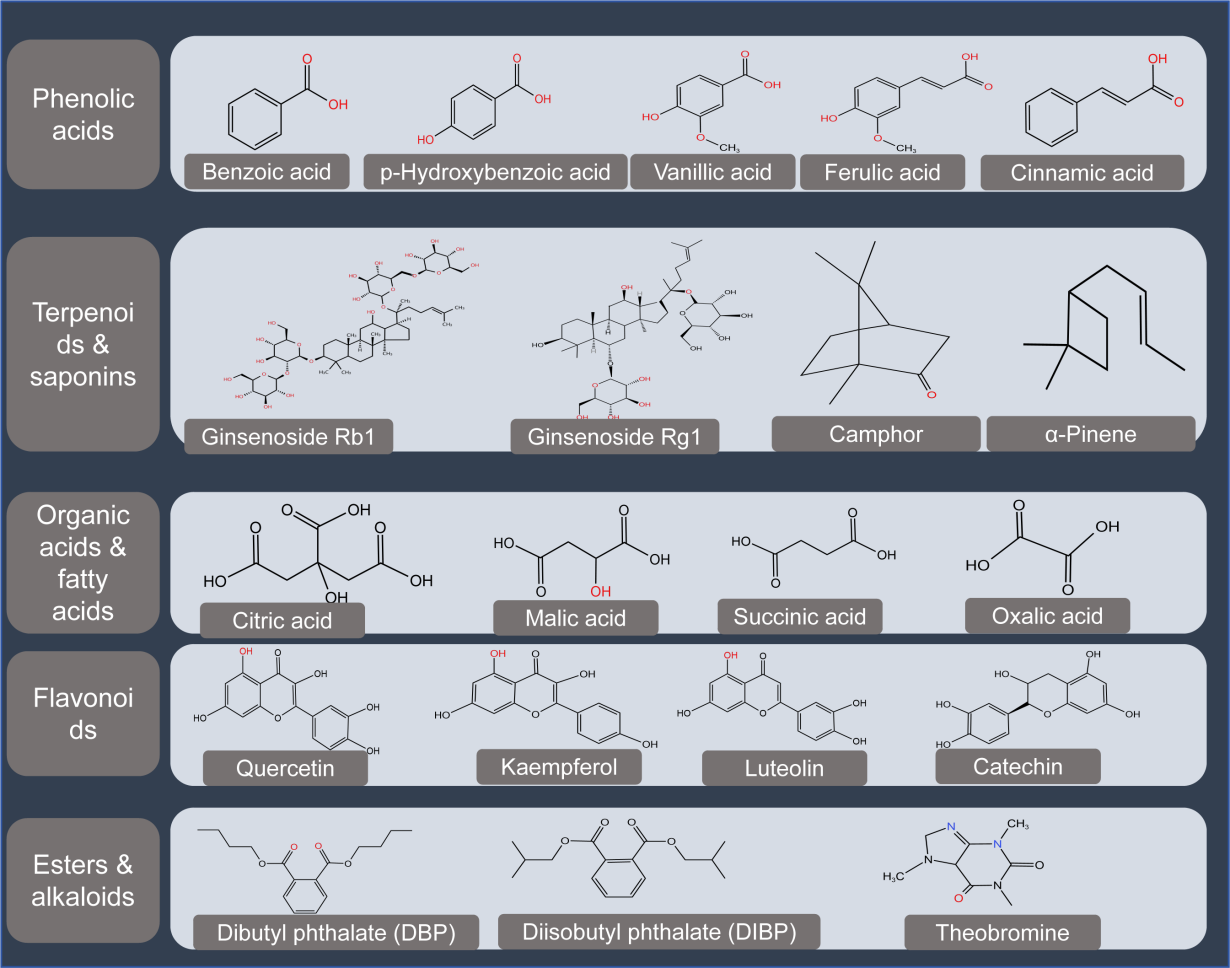
Allelochemicals involved in autotoxicity and obstacles to continuous cropping.

During the continuous cropping of medicinal plants, allelochemicals, a diverse group of secondary metabolites, accumulate in the soil via root exudates or plant residues. In species with root- or underground organ-based production, such as *P. notoginseng*, *P. ginseng*, and *A. membranaceus*, prolonged accumulation triggers continuous cropping disorders (Gao et al., 2024). Compared with conventional agricultural crops, medicinal plants naturally accumulate secondary metabolites with diverse structures and strong bioactivity. Their medicinal active ingredients often have a high degree of overlap with allelochemicals, which is an important characteristic that distinguishes continuous cropping obstacles of medicinal plants from those of common crops (Haq et al., 2025). The major allelochemicals contributing to autotoxicity and rotation-related problems fall into four categories: phenolic acids (e.g., ferulic acid, p-hydroxybenzoic acid, vanillic acid, and protocatechuic acid), flavonoids/polyphenols, terpenoids (saponin triterpenes), and nitrogen-containing compounds such as alkaloids and phenethyl glycosides (**Figure 1**). Most allelochemicals can directly exert phytotoxic effects on host plants by inhibiting root tip cell division and elongation, disrupting cell membrane integrity and ion homeostasis, and triggering reactive oxygen species (ROS) accumulation and membrane lipid peroxidation, ultimately resulting in cell damage or cell death. For example, in the medicinal plant *Panax notoginseng*, the autotoxic saponin Rg1 can induce excessive accumulation of O_2_·^-^ and H_2_O_2_ in root cells, and suppress the ASC and GSH cycles, thereby compromising membrane integrity and leading to root cell death (Yang et al., 2018).

In addition, allelochemicals act as key chemical signals that drive ecological shifts in the rhizosphere. In *Rehmannia glutinosa*, metabolomic and rhizosphere ecological analyses have shown that catalpol, acteoside, and their related compounds are the major active metabolites in both root exudates and rhizosphere soil. Among these, catalpol is more abundant in root exudates and continues to accumulate in the rhizosphere during tuber enlargement. This accumulation is accompanied by significant changes in key rhizosphere taxa, including *Pseudomonas*, *Lysobacter*, and *Fusarium*, suggesting that catalpol functions not only as a potential autotoxic factor but also as an important chemical signal driving rhizosphere ecological shifts (Zhang et al., 2020a). Consistent findings have been reported in classic studies of continuous cropping in *Pseudostellaria heterophylla*, which demonstrated that phenolic acids in root exudates do not release transiently but accumulate gradually with plant growth and development. Under long-term continuous monoculture, the abundance of *Fusarium oxysporum* in the rhizosphere soil increases significantly; further experiments confirmed that root exudates can promote mycelial growth by up to 23.8% and spore production by up to 12.5-fold, directly demonstrating that allelochemical accumulation enhances the fitness of soil-borne pathogens (Zhao et al., 2015). Similar patterns have been documented in *Astragalus membranaceus* continuous cropping systems, where long-term monoculture drives pronounced alterations in the rhizosphere metabolite profile, with 2-aminophenol, quinic acid, tartaric acid, and maleamate identified as candidate inhibitory metabolites. Of these, the first three compounds significantly inhibited the root growth of *A. membranaceus* in a dose-dependent manner, confirming that metabolites accumulated during continuous cropping can directly exert autotoxic effects on host plants (Zhou et al., 2024).

Collectively, existing studies demonstrate that while the dominant allelochemicals vary in structural characteristics across medicinal plant species, their accumulation dynamics and biological consequences exhibit several conserved features. First, these allelochemicals are not released in a transient manner; rather, they are continuously secreted throughout the entire plant growth cycle and progressively accumulate under long-term continuous cropping. Second, they consistently reach higher local concentrations in the rhizosphere than in bulk soil, thus exerting spatially specific regulatory effects. Third, their biological targets include not only the host plant itself but also rhizosphere pathogens and beneficial microorganisms, thereby mediating both direct autotoxic effects on the host and indirect disease-promoting effects via rhizosphere microbial modulation.

### 5.2 Changes in the soil chemical environment caused by continuous cropping

Many studies have shown that with an increase in continuous cropping duration, the accumulation of allelochemicals is often accompanied by soil acidification, redistribution of available nitrogen, and speciation of certain metal elements (Li et al., 2021a; Chen et al., 2023; Zeeshan Ul Haq et al., 2023). Soil acidification is the most prevalent and representative chemical signature of continuous cropping obstacles among these changes. For example, in ginseng continuous cropping systems, soil pH under conventional fertilization regimes can drop to as low as 4.45. Following the application of woody plant-derived biochar or maize straw biochar, the soil pH increased by 0.2–0.3 units, and the relative abundance of pathogenic *Fusarium* decreased by 19% and 35%, respectively. These amendments also improved the complexity of the rhizosphere microbial network and increased crop yield (Liu et al., 2022a). This evidence confirms that soil acidification is not only a core diagnostic indicator of continuous cropping obstacles but is also tightly linked to the formation of a disease-conducive rhizosphere microecosystem.

In addition to soil acidification, shifts in soil nitrogen speciation and the carbon-to-nitrogen (C/N) ratio also drive profound alterations in the rhizosphere microenvironment. In medicinal plant cultivation systems, high rates of nitrogen fertilizer are routinely applied to meet the nutritional demands of belowground medicinal organs. However, under long-term continuous cropping, this fertilization regime frequently results in excessive soil nitrogen accumulation, a decreased soil C/N ratio, accelerated mineralization of soil organic matter, and elevated concentrations of soluble nitrogen and dissolved organic carbon (Wang et al., 2022). When ammonium nitrogen (NH_4_^+^-N) predominates over nitrate nitrogen (NO_3_^-^-N) in the soil, acidification is significantly promoted, denitrification and fermentative metabolic pathways are upregulated, and the relative abundance of pathogenic fungi and facultative anaerobic bacteria increases markedly (Maywald et al., 2023). Conversely, when nitrate nitrogen is the dominant form or organic nitrogen is released in a slow, sustained manner, the rhizosphere environment is more conducive to the colonization of aerobic, oligotrophic beneficial microorganisms (Gu et al., 2020).

Therefore, continuous cropping obstacles are not caused by changes in a single soil chemical factor but rather reflect the combined effects of multiple processes, including soil acidification, nutrient imbalance, and element activation. To address these problems, the rational application of soil amendments, such as biochar, organic fertilizers, and medicinal plant-derived straw, can effectively buffer soil acidification, optimize the soil C/N ratio, reduce the abundance of pathogenic fungi, and enhance the stability of the rhizosphere microbial network, thereby significantly alleviating soil-borne root rot and continuous cropping obstacles in medicinal plants, such as ginseng (Li et al., 2024a; Tian et al., 2021).

### 5.3 Allelochemical-mediated remodeling of the soil microbiome

**Figure 2:**
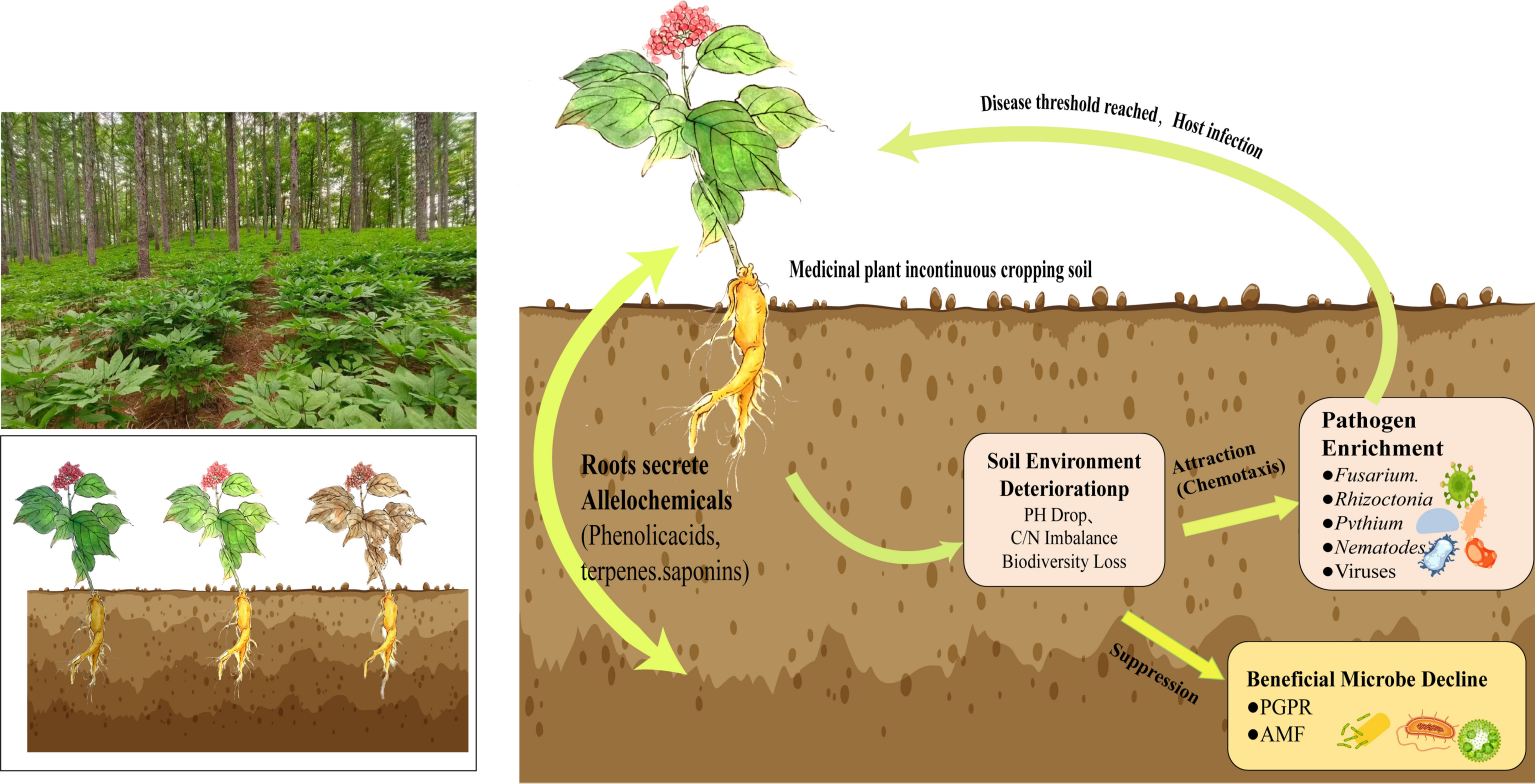
Chemo-biological feedback loop driving soil-borne diseases in continuous cropping systems.

In continuous monocropping systems, allelochemicals not only exert direct autotoxic effects on host plant roots but also act as key ecological filters that drive the remodeling of the rhizosphere microbiome. As these allelochemicals (released from root exudates and decomposing plant residues) progressively accumulate in the soil, they gradually disrupt the inherent rhizosphere microecological balance via differential regulation of the growth, chemotaxis, root colonization, and metabolic activity of rhizosphere microorganisms. This selective filtering process drives a directional shift in the soil microbial community from a disease-suppressive state to a disease-conducive state, which ultimately exacerbates continuous cropping obstacles and the onset of soil-borne diseases (**Figure 2**).

Existing studies have consistently demonstrated that the signature allelochemicals that accumulate during continuous cropping across different medicinal plant species exert strong selective pressure on both pathogenic and beneficial rhizosphere microorganisms. For instance, ginsenosides in the root exudates of *P. ginseng* promote the growth of pathogenic fungi, such as *Pythium* and *Phytophthora*, while suppressing beneficial biocontrol fungi, such as *Trichoderma hamatum* (Nicol et al., 2003). In soils continuously cultivated with *Coptis*, alkaloids, including berberine and coptisine, accumulate to high levels and strongly inhibit some fungal species; however, they have a limited impact on relatively highly tolerant pathogens (Alami et al., 2020). Pathogen enrichment is accompanied by a decline in the abundance of beneficial microbes and their key functions. Long-term continuous cropping of *Astragalus* reduced rhizosphere and endophytic microbial diversity, coinciding with the accumulation of phenolic acids and flavonoid allelochemicals. Crop rotation or organic amendments can partially restore the abundance of plant growth-promoting rhizobacteria and arbuscular mycorrhizal fungi and reduce root rot incidence (Zhou et al., 2024). Similar trends have been observed in *P. notoginseng*, where restoration of microbial communities through optimized water management or application of bio-organic fertilizers alleviates continuous cropping disorders and root rot (Bao et al., 2022; Guo et al., 2022).

Furthermore, allelochemical-driven changes involve shifts in microbial community composition and systematic restructuring of the rhizosphere’s microbial interaction networks. In healthy soils, bacteria, fungi, and other functional microorganisms typically form tightly connected co-occurrence networks with complex interaction patterns. Higher node and edge numbers, greater average connectivity, and stronger modularity help maintain community stability and ecological buffering capacity. However, in the context of continuous cropping obstacles, the accumulation of allelochemicals and enrichment of pathogens often lead to reduced network size, fewer inter-taxa connections, loss of key hub taxa, and an imbalance between positive and negative microbial interactions, resulting in decreased network complexity and stability. In particular, once cooperative interactions dominated by beneficial microorganisms are weakened, the microbial community’s resistance to external stress and pathogen invasion declines, and the soil microecology becomes more prone to shifting toward a fragile, disease-conducive state (Dong et al., 2022). For example, in continuously cultivated *Panax quinquefolius*, the number of edges in the rhizosphere bacterial co-occurrence network decreased from 6760 to 6065 with an increase in continuous cropping years, and network density decreased from 0.343 to 0.314, whereas the modularity index increased from 0.506 to 0.663. Based on these results, the authors concluded that continuous cropping reduced the complexity and stability of the co-occurrence network and caused the bacterial community to become more fragmented (Song et al., 2025).

The results indicate that the key to alleviating continuous cropping obstacles is to rebuild the overall structure and function of the rhizosphere microbiome, rather than simply suppressing a single pathogen. More importantly, allelochemicals drive a negative feedback loop that worsens continuous cropping obstacles: the accumulation of allelochemicals enriches pathogens and reduces beneficial microbes, which further damages host roots and causes them to release more allelochemicals. Ultimately, this vicious cycle of “allelochemical accumulation–microbial imbalance–pathogen enrichment–host damage–greater allelochemical release” pushes the soil from a healthy state toward an increasingly disease-conducive state.

## 6 Immunometabolic basis of host susceptibility

### 6.1 Continuous cropping stress reshapes the host pre-immune state

In continuous cropping systems of medicinal plants, hosts do not encounter soil-borne pathogens under stable conditions. Instead, they are preconditioned into a persistently disturbed immune state by the combined effects of allelochemical accumulation, changes in soil physicochemical properties, and rhizosphere microecological imbalances. This long-term stress often disrupts root redox homeostasis, accompanied by an antioxidant imbalance, increased membrane lipid peroxidation, and higher metabolic maintenance costs, thereby weakening the host’s capacity to respond to subsequent biotic stress (He et al., 2022b). Simultaneously, changes in root exudates can further alter the direction of microbial assembly, leading to coordinated shifts in the host immune status, metabolic background, and rhizosphere ecology. For example, continuous cropping of *Rehmannia glutinosa* can cause oxidative damage in root tips and promote the spread of *Fusarium oxysporum* in the rhizosphere through exudate-mediated effects (Li et al., 2023). The accumulation of active exudates, such as catalpol, is accompanied by marked changes in key taxa, including *Pseudomonas*, *Lysobacter*, and *Fusarium* (Zhang et al., 2020a), indicating continuous coupling among oxidative load, metabolic reprogramming, and shifts in microbial assembly. This is particularly important in medicinal plants because both defense and quality rely heavily on the dynamic allocation of specialized metabolites, such as phenylpropanoids, terpenoids, and alkaloids. Therefore, the chronic low-level stress caused by continuous cropping can reprogram resource allocation in advance, making the host more prone to delayed defense, insufficient responses, or metabolic mismatch during subsequent infection.

### 6.2 From PTI and ETI to ROS, Ca²^+^, and nuclear signaling: the mechanisms underlying basal immunity disruption

Early pathogen recognition in plants mainly depends on a two-tiered immune system. The first is pattern-triggered immunity (PTI), which is mediated by plasma membrane pattern recognition receptors that detect pathogen- and damage-associated molecular patterns. The second is effector-triggered immunity (ETI), which is mediated by intracellular NLR receptors that recognize pathogen effectors or the host disturbances they cause. These two layers are not separate; rather, they are highly coupled through Ca²^+^ signaling, ROS bursts, MAPK/CDPK cascades, and transcriptional reprogramming, and together, they determine the strength of the immune output (Mansoor et al., 2022) (Bogeski et al., 2011).

During PTI, recognition of PAMPs or MAMPs by PRRs rapidly triggers membrane depolarization, Ca²^+^ influx, an RBOHD-mediated ROS burst, and MAPK cascade amplification (Bigeard et al., 2015). ETI usually induces stronger and more sustained Ca²^+^ signaling, ROS accumulation, and defense gene expression, and is therefore often regarded as an amplified immune state rather than a completely independent parallel pathway (Ngou et al., 2021). Ca²^+^ and ROS are widely recognized as shared core signaling nodes linking PTI and ETI. Ca²^+^ flux activates calcium-dependent protein kinase and calmodulin-related pathways, promoting NADPH oxidase-mediated ROS production. ROS, in turn, further amplifies Ca²^+^ signaling and contributes to cell wall reinforcement, establishment of an antimicrobial environment, and initiation of programmed cell death (Wang and Luan, 2024). Under continuous cropping, long-term oxidative stress can weaken the host’s ability to recognize newly encountered pathogen signals and disrupt the spatial and temporal precision of the Ca²^+^-ROS positive feedback loop, making it difficult to coordinate cell wall reinforcement, defense gene induction, and timely establishment of a local antimicrobial environment (Yu et al., 2024). In addition to PAMPs, DAMPs should be considered in continuous cropping systems. Under allelopathic stress, mechanical injury, and pathogen attack, root tissues continuously release danger signals, such as cell wall fragments, peptide signals, and extracellular ATP (eATP). Among these, eATP has been identified as an important plant DAMP that is sensed by P2K1/DORN1 and P2K2, and can trigger Ca²^+^ influx, ROS bursts, and defense-related transcriptional responses. Notably, eATP signaling is not amplified without limits; its strength and duration are constrained by receptor regulation and negative feedback. This suggests that under continuous cropping, persistent tissue damage and autotoxic stress may maintain DAMP signaling over time, thereby shifting defense from a transient warning response to chronic imbalance (Choi et al., 2014). More importantly, eATP signaling is not unlimited. For example, in *Arabidopsis*, eATP released by local injury requires P2K receptors to trigger a systemic ROS wave, whereas in root disease systems, P2K1/DORN1 can enhance resistance to *Rhizoctonia solani* (Myers et al., 2022). Simultaneously, P2K1 is dynamically regulated by S-acylation, and the timing of its autophosphorylation and protein degradation is tightly controlled, indicating clear negative-feedback regulation over the intensity and duration of eATP signaling (Kumar et al., 2020). This suggests that persistent tissue damage and autotoxic stress under continuous cropping may sustain DAMP signaling, thereby aggravating immune imbalance. Another key step in basal immunity is signal transmission into the nucleus. Whether initiated by PRRs or NLRs, immune signaling must ultimately pass through nuclear transcriptional regulatory networks involving MAPKs, CDPKs, CaM/CMLs, NPR1, and CBP60/SARD1 to convert membrane and cytoplasmic alarm signals into expression programs for defense, metabolic, and cell wall remodeling genes (Mou et al., 2003; Li et al., 2016). Disturbance of ROS or Ca²^+^ homeostasis, energy deficiency, and changes in chromatin state caused by continuous cropping stress may all interfere with this process, from signal entry into the nucleus to transcriptional output.

### 6.3 Programmed cell death

Programmed cell death (PCD) is an important component of plant immunity, and timely, moderate cell death helps restrict pathogen spread and amplifies local defense signals (Coll et al., 2011). Recent studies have further shown that immune-related cell death in plants is not a single mode. Among these forms, ferroptosis-like death, which involves ROS, Ca²^+^, iron homeostasis, and lipid peroxidation, has attracted increasing attention (Shen and Naqvi, 2024). However, in continuous cropping systems of medicinal plants, the role of PCD shows a clear threshold effect. Moderate local cell death can isolate infection sites, release DAMPs, and promote defense amplification (Tanaka and Heil, 2021), whereas under long-term high ROS levels, enhanced lipid peroxidation, and disrupted iron homeostasis in root tissues, PCD may shift from a controlled immune output to tissue damage (Dangol et al., 2019). In root rot and tissue-macerating diseases, excessive or mistimed cell death can increase tissue softening and nutrient leakage, thereby creating favorable conditions for the spread of soil-borne fungi, bacteria, and saprophytes. For example, *Pectobacterium brasiliense*, a typical soft rot pathogen, can cause tuber softening through cell wall-degrading enzyme-mediated tissue maceration and host cell death, while the nutrient-rich microenvironment formed by leakage of cellular contents further promotes pathogen expansion (Davidsson et al., 2013). Therefore, under continuous cropping, PCD should not be regarded as a marker of enhanced resistance but rather as a key turning point that determines whether the host maintains resistance or becomes more susceptible.

### 6.4 Hormone, transcription, and metabolism coupled reprogramming

#### 6.4.1 Reorganization of hormone networks drives the reallocation of defense resources

After immune signaling is amplified, plants must reallocate their limited resources to growth, repair, and disease resistance. This process is regulated not only by classical hormone networks, such as SA, JA, ET, and ABA, but also depends heavily on energy status and the reprogramming of central metabolism.

SA drives PR gene expression and the establishment of systemic acquired resistance (SAR) through NPR1 and its associated nuclear regulatory network (Mou et al., 2003), whereas NHP participates in the spread of systemic immune signals (Yildiz et al., 2021). JA-Ile activates transcriptional programs, such as MYC2, through the COI1-JAZ module and often acts together with ET through the EIN3/EIL1-ERF branch to strengthen defense against necrotrophic pathogens (Zhu et al., 2011). Under continuous cropping, persistent allelopathic stress and oxidative pressure often increase ABA levels, which can reshape the defense priority through antagonistic interactions with SA and JA-ET signaling, thereby shifting the host toward adaptation to abiotic stress rather than targeted disease resistance (Jia et al., 2024).

Metabolic constraints after immune activation should not be overlooked. ATP is not only the intracellular energy currency but also an important source of extracellular danger signals. Therefore, increased eATP often indicates tissue damage, whereas insufficient intracellular ATP supply can limit the sustained operation of defense metabolism, ion transport, membrane repair, and transcriptional reprogramming (Tanaka et al., 2014). In other words, under continuous cropping, hosts facing pathogen attacks often experience a contradictory state of enhanced immune signaling and restricted energy supply (Wang et al., 2023b). Recent studies have shown that TCA cycle intermediates, such as citrate, malate, succinate, fumarate, and α-ketoglutarate, are not merely metabolic intermediates, but also participate in redox balance, stress signaling, carbon-nitrogen coordination, substrate supply for epigenetic regulation, and rhizosphere chemical communication (Zhang et al., 2023b). For example, citrate and malate are involved not only in mitochondrial metabolism and cytosolic carbon reallocation but also commonly act as major root-secreted organic acids that influence metal ion activity and microbial recruitment (Lee et al., 2021). In particular, the citrate-acetyl-CoA axis can affect the supply of substrates for histone acetylation (Chen et al., 2017), thereby indirectly regulating the chromatin accessibility of immune-related genes. Overall, under continuous cropping, immunity and metabolism do not act in a simple sequence but are mutually dependent. Immune responses reshape central metabolism, whereas the metabolic state determines whether immunity can be maintained, amplified, and accurately executed.

Consequently, the rewiring of hormonal networks and central metabolism under continuous cropping not only determines disease progression but also redirects the accumulation patterns of bioactive compounds, constituting a key physiological basis for the coordinated regulation of disease resistance and medicinal quality. For example, in *Panax notoginseng*, autotoxic stress induced by saponin accumulation under continuous cropping triggers root metabolic reprogramming, activates the phenylpropanoid pathway and α-linolenic acid metabolism, and reshapes root exudate profiles. These changes facilitated the recruitment of the beneficial bacterium *Burkholderia* sp. B36 and enhanced the host survival. This evidence demonstrates that shifts in bioactive compound metabolism can simultaneously modulate rhizosphere defense interactions and determine the host health status (Deng et al., 2023).

#### 6.4.2 Transcription factor modules link hormone signaling to metabolic reprogramming

Alterations in phytohormone networks and central metabolic status are ultimately translated into distinct defense and metabolic outputs via genome-wide transcriptional regulatory programs. This regulatory layer is especially critical in medicinal plants, and different transcription factor families together form a key regulatory network linking immune signaling, specialized metabolism, and tissue homeostasis. They determine whether limited resources are preferentially directed toward PR protein expression, cell wall reinforcement, ROS regulation, or quality-related metabolic pathways, such as phenylpropanoids, terpenoids, and alkaloids.

Among them, the NPR1-TGA-WRKY module mainly mediates SA-related defense output and coordinates defense gene expression to establish systemic acquired resistance. Previous studies have shown that WRKY members, such as EbWRKY44, PeWRKY30, and SfWRKY29, can positively regulate flavonoid and phenylpropanoid metabolism, thereby enhancing the accumulation of defense-related metabolites (Zhong et al., 2025). This suggests that the SA pathway not only drives the expression of classical PR proteins but also channels immune signals into metabolic networks through the WRKY module. The MYC2/bHLH module is an important interface through which JA signaling is converted into specialized metabolism and stress responses. It affects not only terpene biosynthesis but also works with MYB modules to regulate quality-related metabolites, such as phenolic acids (Shen et al., 2016). This case shows that the JA/MYC2 module not only contributes to disease resistance but also directly determines the formation of active compounds in medicinal plants, making it a typical example of immunity-quality coupling. The ERF/ORA59 module is closely involved in ET/JA-related cell wall reinforcement, glycosylation regulation, and defense against necrotrophic pathogens. For example, in *Morinda officinalis*, the ethylene-responsive factor MoERF10 can regulate glycosylation-related metabolism and promote cell wall lignification, thereby enhancing resistance to stem basal rot (Chen et al., 2023d). Simultaneously, the MYB family plays a central role in the phenylpropanoid pathway and in lignin and flavonoid biosynthesis (Geng et al., 2020), whereas transcription factors such as NAC and bZIP are more involved in cell wall remodeling, stress responses, and the maintenance of tissue homeostasis (Guan et al., 2019). In other words, these transcription factors do not act independently but together determine whether the immune output can be converted into appropriate metabolic and structural responses.

#### 6.4.3 The target gene execution layer determines defense metabolism, quality formation, and rhizosphere regulation

Following signal integration by transcription factors, the final outcomes of host defense and quality formation depend on the target gene execution layer. These genes were categorized into four types: synthesis, modification, transport/sequestration, and structure/detoxification. Together, these factors determine the effective synthesis, modification, transport, and targeted accumulation of metabolites. This forms the ultimate execution interface linking immune signaling, tissue defense, active compound formation and microbiome regulation.

Modification targets (e.g., *UGT, OMT, AT*, and *CYP*) remodel metabolite structures, thereby affecting their stability and medicinal quality. In *Arabidopsis*, the pathogen-inducible glycosyltransferase UGT73C7 glycosylates upstream substrates, such as p-coumaric acid and ferulic acid, redirects flux toward hydroxycinnamate and coumarin branches, and is coupled to NLR-mediated immune activation (e.g., SNC1) (Huang et al., 2021). Such modification-related genes can also tune immune strength and duration by altering the activities of small molecules (Holmes et al., 2021). In some cases, modified products themselves function as immune effectors; pathogen-induced UGT73C3/4 glycosylate the lignan pinoresinol and promote ROS production and callose deposition, thereby enhancing resistance, consistent with the feedback amplification of defense responses (Zhao et al., 2025). In *Isatis indigotica*, OMT-mediated methylation increases flavonoid stability and shapes medicinal quality and profiles of bioactive compounds (Tan et al., 2022); acyltransferases (Murayama et al., 2021) and CYP450 monooxygenases similarly influence product diversification, as exemplified by CYP71AV1 in *A. annua* (Shen et al., 2016) and CYP98A14 in *S. miltiorrhiza* (Zhou et al., 2021). Transport- and sequestration-related targets (e.g., ABCG, MATE, and SWEET) determine whether metabolites are secreted into the rhizosphere or compartmentalized in vacuoles/cell walls, thereby linking host metabolism to rhizosphere suppression of pathogens and microbiome recruitment. Antimicrobial metabolites often require transporters to exert their activities in the rhizosphere (Fourcroy et al., 2014). For instance, the MATE alkaloid transporter Nt-JAT1 localizes to the tonoplast and mediates nicotine transport into vacuoles, thereby sequestering a highly toxic defense compound and balancing defense efficacy with the control of autotoxicity (Morita et al., 2009).

Structural and detoxification targets support barrier integrity and stress tolerance. Cell wall-remodeling genes, such as *PME/PMEI* (Bai et al., 2025), *XTH* (Zhan et al., 2025), and *EXP* (Liu et al., 2025), and callose biosynthesis (Ellinger et al., 2013) enhance resistance to penetration. In contrast, antioxidant systems (superoxide dismutase, catalase, and ascorbate peroxidase) restrain ROS to preserve signaling while preventing sustained oxidative damage (Sheng et al., 2022). Glutathione S-transferase regulates glutathione homeostasis and ROS detoxification, clears lipid peroxides during infection (Otulak-Kozieł et al., 2022), and directly conjugates fungal toxins with glutathione to generate detoxified products that are further processed or compartmentalized (Michlmayr et al., 2025).

The ultimate output of host immunity extends beyond intracellular defense to manifest at the rhizosphere microecosystem level through chemical communication. Root exudates comprise sugars, organic acids, amino acids, phenolic acids, coumarins, flavonoids, terpenoids, and other defense-associated metabolites, which serve as microbial carbon sources and signaling cues and as key ecological mediators for host-driven recruitment of beneficial microbes and exclusion of pathogens (Sasse et al., 2018). Therefore, changes in immune status are often accompanied by shifts in the root exudate profile, which in turn further redirect microbiome assembly. Under continuous cropping, this process often appears as a continuous chain of immune output shifts, altered metabolic secretion, and microbial community restructuring. When the host can mount a timely and moderate defense response, rhizosphere exudation is more favorable for maintaining a disease-suppressive microecology and promoting the colonization of plant growth-promoting or induced resistance-related microbial groups (Yang et al., 2023a). In contrast, when continuous cropping leads to basal immune imbalance, excessive programmed cell death, or hormone and metabolism reprogramming biased toward damage responses, roots may release excessive amounts of certain phenolic acids, organic acids, and tissue degradation products. This can increase pathogen chemotaxis, attachment, and nutrient utilization while weakening the selective advantage of beneficial microorganisms (Wu et al., 2017). In medicinal plants, specialized rhizosphere metabolites often play dual roles in defense and quality formation. Therefore, changes in their secretion not only affect disease progression but also feedback on the microecological environment that supports the formation of medicinal quality (Luo et al., 2020). Taken together, host immunity is not an isolated intracellular event but continuously exports ecological effects to the rhizosphere through metabolic and secretory reshaping. Immune status affects metabolism, metabolism reshapes the microbial community, and the microbial community in turn feeds back on host immunity and metabolic homeostasis. This closed loop should be regarded as a key ecological mechanism underlying the development of susceptibility in continuously cropped medicinal plants.

In summary, hormone networks determine how defense resources are allocated, transcription factor modules determine how metabolic and structural defense programs are initiated, and target genes of different functional classes determine whether these programs can be effectively executed, ultimately influencing pathogen suppression, the accumulation of bioactive compounds, and the reshaping of rhizosphere microecology.

### 6.5 Key regulatory dimensions in the development of susceptibility: dose thresholds, critical time windows, and genotypic differences

Host susceptibility is not determined by a single factor but is jointly regulated by three core dimensions: dose threshold, critical time window, and genotypic differences. First, the dose threshold shapes the immune outcome (Jalal et al., 2021). Low-level stimulation can trigger defense priming; however, once allelopathic stress and damage signals accumulate beyond a threshold, immunity may shift from pre-activation to chronic imbalance, increasing the risk of infection. Second, the critical time window determines defense effectiveness (Silvia Sebastiani et al., 2017). The first 24–48 h after infection are crucial for activating defense processes, such as signal transduction and metabolic reprogramming. If continuous cropping stress delays defense during this window, even a strong later response is often unable to reverse the disease progression. Finally, genotypic differences determine the host’s buffering and adaptive capacities (Zhang et al., 2023a). Highly resistant genotypes have stronger redox buffering, resource allocation, and rhizosphere-stabilizing abilities, whereas highly susceptible genotypes are more likely to show delayed defense, metabolic imbalance, and microbial mismatch under the combined pressure of continuous cropping and pathogen attack (Liu et al., 2022b).

**Figure 3:**
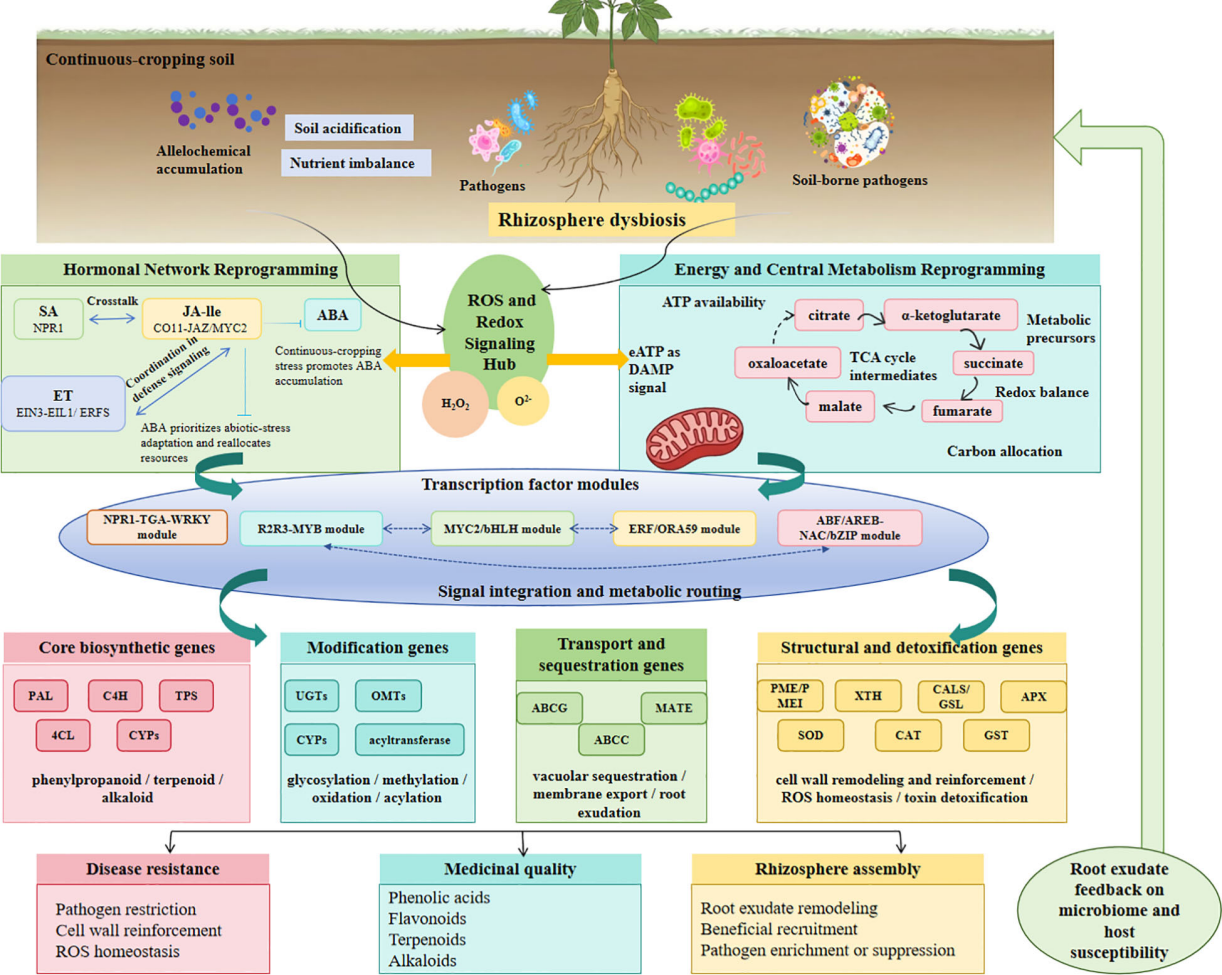
Model for the coordinated regulation of immunity, metabolism and rhizosphere in medicinal plants under continuous cropping stress.

In summary, the development of host susceptibility under continuous cropping is a dynamic process initiated by environmental pre-disturbance, amplified through basal immune imbalance and shifts in the PCD threshold, and jointly shaped by hormone, transcription, and metabolism reprogramming, together with rhizosphere microecological restructuring. Dose thresholds, critical time windows, and genotypic differences determine whether this process ultimately leads to the maintenance of resistance or aggravation of susceptibility (**Figure 3**).

## 7 Development of microbiome-guided integrated management strategies

**Figure 4:**
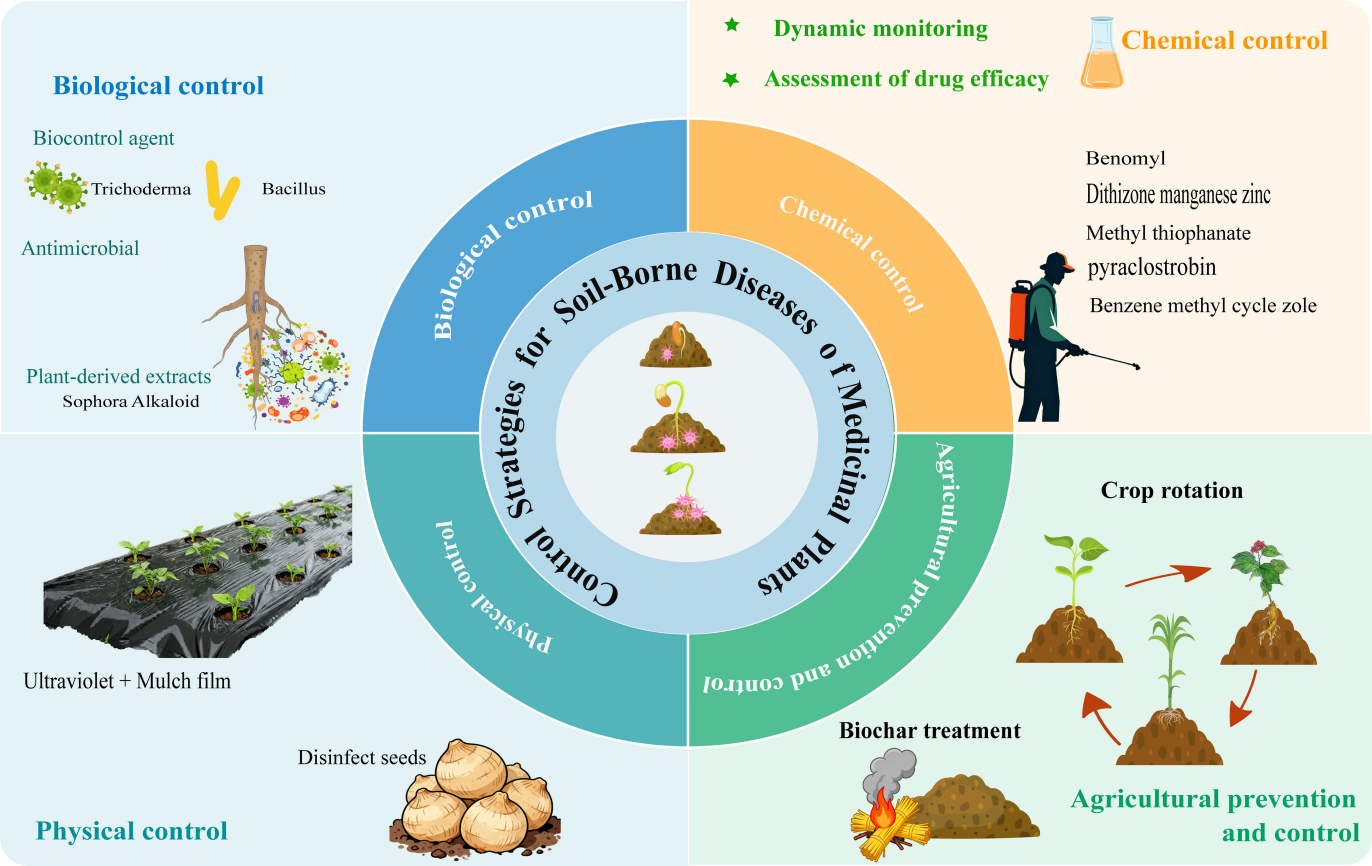
Methods for controlling soil-borne diseases in medicinal plants.

Various control strategies for soil-borne diseases in medicinal plants are available (**Figure 4**); however, chemical fungicides and a limited set of antagonistic agents have long been the main approach, suffering from inconsistent efficacy and compromised safety of medicinal constituents. Under the pressure of soil-borne pathogens, plants function as an integrated system with rhizosphere and endophytic microbiota, and health or disease is shaped by the structure of this community (Spooren et al., 2024). Accordingly, the suppression of soil-borne pathogens often depends on microbiome-mediated processes. In medicinal plants, shifts in these microbial communities affect disease incidence and yield and can influence the accumulation and biotransformation of medicinal compounds.

Although biological agents and soil amendments have shown potential for controlling soil-borne diseases in medicinal plants, their efficacy is highly context-dependent and cannot be generalized across host species, soil types, or pathogen complex systems. The reported outcomes vary greatly. In strain screening experiments, *in vitro* inhibition rates are often relatively high, whereas reductions in disease incidence under greenhouse or field conditions are usually much more limited, and these two types of results are not directly comparable. In addition, the effectiveness of a given intervention can vary with soil pH, organic matter content, salinity level, indigenous microbial communities, pathogen inoculum pressure, and whether the disease is driven by a single pathogen or a complex infection system. Therefore, when evaluating the performance of management measures, it is necessary to explicitly consider their potential trade-offs, including unintended disturbances to the native microbiome and possible effects on medicinal quality traits and the accumulation of specialized secondary metabolites.

### 7.1 Plant growth-promoting bacteria

Biocontrol bacteria have shown potential in the management of soil-borne diseases in medicinal plants; however, their efficacy is highly strain-specific and is strongly influenced by crop species, soil conditions, and the composition of pathogen communities. A wide range of biocontrol bacteria has been reported to control soil-borne diseases in medicinal plants. For example, *Bacillus velezensis* NT35 alleviated rust root symptoms in ginseng, promoted plant growth, and improved the structure of the rhizosphere microbial community (Li et al., 2024e). Similarly, *Brevundimonas terrae* SZ-22, isolated from the ginseng rhizosphere, showed an inhibition rate of 83.7% against *Alternaria panax* under experimental conditions (Sun et al., 2018). PGPB can protect plants via multiple mechanisms, including antibiotic biosynthesis, competition for nutrients and colonization niches, and siderophore-mediated iron chelation.

However, the field performance of PGPB in disease control is inconsistent, and their effects vary with soil, crops, and climatic conditions. The effectiveness of PGPB depends largely on their ability to colonize under field conditions and compete with the native microbiome (Rilling et al., 2018).In addition, when faced with complex disease networks caused by combined fungal and bacterial infections, intervention with a single strain is often limited (Niu et al., 2020). Existing studies have shown that PGPB are influenced by the soil environment, host genotype, and application method. Although they can suppress target pathogens, they may also alter the composition and assembly of rhizosphere and endophytic microbial communities, and such changes are not necessarily beneficial to medicinal plants (Etesami, 2025). For example, PGPB can increase tuber yield and polysaccharide accumulation in *Pseudostellaria heterophylla* only under elevated CO_2_ conditions (Ng et al., 2024). Therefore, when applying exogenous PGPB in the cultivation of medicinal plants, attention should be paid not only to their growth-promoting and disease-suppressive potential but also to their possible effects on microecology and metabolism related to medicinal quality.

### 7.2 Synthetic microbial communities

Single-strain inoculants often perform well under controlled conditions; however, their efficacy in the field is frequently constrained by soil physicochemical properties and environmental variability. SynComs comprise cultivable microorganisms isolated from ecological niches, such as rhizosphere soil or plant roots, which are artificially combined in specific ratios to achieve functional objectives, including disease suppression, growth promotion, and enhancement of medicinal-plant quality (Shayanthan et al., 2022).

Two main strategies are used to develop SynComs. In the top-down approach, disease-suppressive or growth-promoting communities are first identified in the rhizosphere of resistant cultivars, and the key members are then isolated through reductionist screening and reassembled into simplified consortia. For example, a simplified microbial community screened from beneficial microbes in the rhizosphere of *Astragalus* significantly reduced root rot incidence (Li et al., 2021b). In the bottom-up approach, the desired community function is defined *a priori*; candidate strains are selected from culture collections based on traits such as antagonism, siderophore production, or phosphate solubilization, and stable consortia are obtained through iterative assembly and performance screening. Similarly, a synthetic community jointly constructed from bacterial and fungal strains in *Atractylodes macrocephala* was used to control root rot, reducing the abundance of *Fusarium* in the rhizosphere by 62% and increasing the proportion of beneficial soil microbes by 25% (Luo et al., 2023).

Compared with single-strain formulations, the advantage of SynCom lies in that its intervention target is no longer limited to suppressing a single pathogen but instead aims to regulate multiple processes simultaneously, including pathogen pressure, resource competition, rhizosphere colonization, and host responses. Therefore, it is better suited to address the common problems of multi-pathogen co-infection and rhizosphere imbalance in the continuous cropping systems of medicinal plants. However, the effectiveness of SynComs does not depend on simply increasing the number of strains but rather on their ecological fitness in the target rhizosphere environment and the controllability of interactions among their members. In other words, a well-designed SynCom should not only be successfully established in complex native communities but also maintain relatively stable cooperation among its members so that its functions are continuously expressed.

Recent studies increasingly view SynComs as designable ecological interventions (Northen et al., 2024) rather than as an empirical mixture of strains. Related studies have shown that providing SynCom with selectable nutritional niches can improve its expansion and persistence in complex communities (Čaušević et al., 2024). Similarly, data-driven methods, such as machine learning, have begun to be used for candidate strain prioritization, inference of cooperative combinations, and prediction of colonization outcomes (Emmenegger et al., 2023). With the development of technologies such as barcode tracking, the contribution of individual strains to rhizosphere colonization and their interaction effects are becoming quantitatively measurable (Ordon et al., 2024). This means that SynCom development is shifting from repeated trials and errors to a design strategy of “predict first, then screen,” which may improve the efficiency of translation from laboratory validation to field applications.

Overall, SynCom provides a more systematic strategy than single-strain products for microbiome-based interventions against soil-borne diseases in medicinal plants. However, further application requires a deeper evaluation of field stability, ecological safety, host specificity, and overall effects on medicinal quality.

### 7.3 Formulation and carrier technologies for enhancing microbiome interventions

The main function of carrier and formulation technologies is to improve the delivery efficiency, survival, colonization rate, and ecological competitiveness of the introduced beneficial microbes in the rhizosphere, thereby enhancing their disease-suppressive and growth-promoting effects. Existing studies have shown that materials such as hydrogels, polysaccharide-based carriers, seed coatings, and biochar can improve the stability of microbiome-based interventions by extending the activity window of viable microbes, creating local microhabitats, or promoting microbial cooperation (Feng et al., 2025). For example, encapsulating a three-strain PGPB consortium in hydrogel capsules and applying it with compost significantly improved crop growth and nutrient uptake (Cruz-Barrera et al., 2025). Likewise, salicylic acid nanoparticles (SA-NPs) and glycyrrhizate nanoparticles (GAS-NPs) can alleviate tomato wilt, although high doses of SA-NPs significantly reduced rhizosphere bacterial abundance and altered the structure of culturable communities (Shoala et al., 2025). In recent years, nano-enabled RNA biopesticides, stimulus-responsive nanomaterials, and porous carbon-based materials have further expanded this field, providing new tools for targeted pathogen suppression and beneficial microbe delivery. For instance, LDH nanosheets can stabilize and slowly release dsRNA, allowing sustained suppression of key pathogen genes for more than 60 days in the tomato soil-borne *Fusarium* pathosystem (Mohamed and Khamis, 2021). Porous carbon-based materials can improve the survival and rhizosphere persistence of beneficial microbes through their high surface area and pore structure, while also enhancing plant disease resistance through the reassembly of root exudates and microbial community networks (Chen et al., 2023b).

However, these technologies cannot function independently in specific soil contexts. Their performance remains jointly constrained by factors such as carrier composition, moisture content, soil pH, *in situ* microbial competition, and host root exudation patterns. In addition, the effects of these new materials on quality-related metabolism in medicinal plants, field residues, and rhizosphere processes require further evaluation.

### 7.4 Microecological restructuring driven by agronomic regulation and physicochemical amendment

Obstacles to the continuous cropping of medicinal plants promote the accumulation of autotoxic compounds and intensify allelopathic effects, thereby disrupting the microecological balance in the soil and weakening the disease resistance potential of the host. Crop rotation improves the physicochemical properties of soil, increases the microbial abundance and diversity of beneficial taxa, disrupts the life cycles of soil-and air-borne pathogens, and alleviates the deleterious effects of crop-derived autotoxic compounds (Woo et al., 2022). For instance, continuous cultivation of American ginseng reshapes soil bacterial and fungal communities, notably increasing the relative abundance of the pathogenic genus *Fusarium* while reducing the abundance of certain beneficial microorganisms (Li et al., 2024c). As many pathogens exhibit host specificity, medicinal crops can be rotated with non-host species (e.g., grasses or legumes) for 2–3 years to limit pathogen buildup in soil. For instance, the rotation of ginseng with *Perilla frutescens* and alfalfa significantly improves soil nutrient status (e.g., organic matter content and urease activity) in continuous cropping systems and reduces the abundance of *Ilyonectria* (Bian et al., 2023). After three years of corn rotation, American ginseng showed increased root biomass, accompanied by a marked enhancement in the relative abundance of the beneficial genus *Arthrobacter* and a reduction in the incidence of root diseases (Jiao et al., 2019). However, the regulatory effects of different crop rotation schemes on different pathogen complexes are inconsistent, and rotation is better regarded as a context-dependent intervention. For pathogens that produce dormant structures with strong environmental tolerance, a short-term rotation of two–three years is often insufficient for complete eradication. For example, in American ginseng, the negative effects of continuous cropping were not fully eliminated even after ten years of rotation (Li et al., 2021a). In addition, if certain leguminous crops, such as vetches and clovers, are selected for rotation, they may improve soil nitrogen status but can also serve as alternative hosts for root-knot nematodes or *Rhizoctonia*. This may increase the risk of root galling, seedling blight, or root rot in the following crop, thereby promoting cross-crop disease transmission (Timper et al., 2006).

In addition to rational crop rotation, which reduces the incidence of soil-borne diseases in medicinal plants, the application of physicochemical amendments can be used to suppress such diseases. The amendment of continuously cropped ginseng soil with corn-stover-derived biochar decreased the relative abundance of *Fusarium*, reduced root rot incidence in ginseng by 35%, and increased yield (Liu et al., 2022a). Biochar application to continuously cropped *P. notoginseng* increases soil pH, enhances nutrient availability, and promotes microbial diversity, thereby improving plant survival (Zhao et al., 2022a). Similar beneficial effects have been documented in continuously cropped *Panax notoginseng* (Zhao et al., 2022a).

The effects of these measures may vary with medicinal plant species, application rate, soil texture and pH, environmental stress, and the composition of the pathogen complex. For example, the application of high doses of biochar in alkaline soils or heavy metal-contaminated soils may reduce kiwifruit yield, while lower Fe content can aggravate iron deficiency chlorosis (Sorrenti et al., 2016). In addition, incompletely composted materials may carry the risk of introducing opportunistic exogenous pathogens. At the same time, although these measures may suppress disease occurrence, they do not necessarily improve medicinal quality or the accumulation of bioactive compounds. Therefore, agronomic regulation and the application of soil amendments must be based on a precise analysis of soil background conditions to achieve a balance between plant growth status and medicinal quality in medicinal plants.

### 7.5 Quantitative strategies to coordinate disease control and medicinal quality maintenance in pathogen-environment-host interaction systems

**Figure 5:**
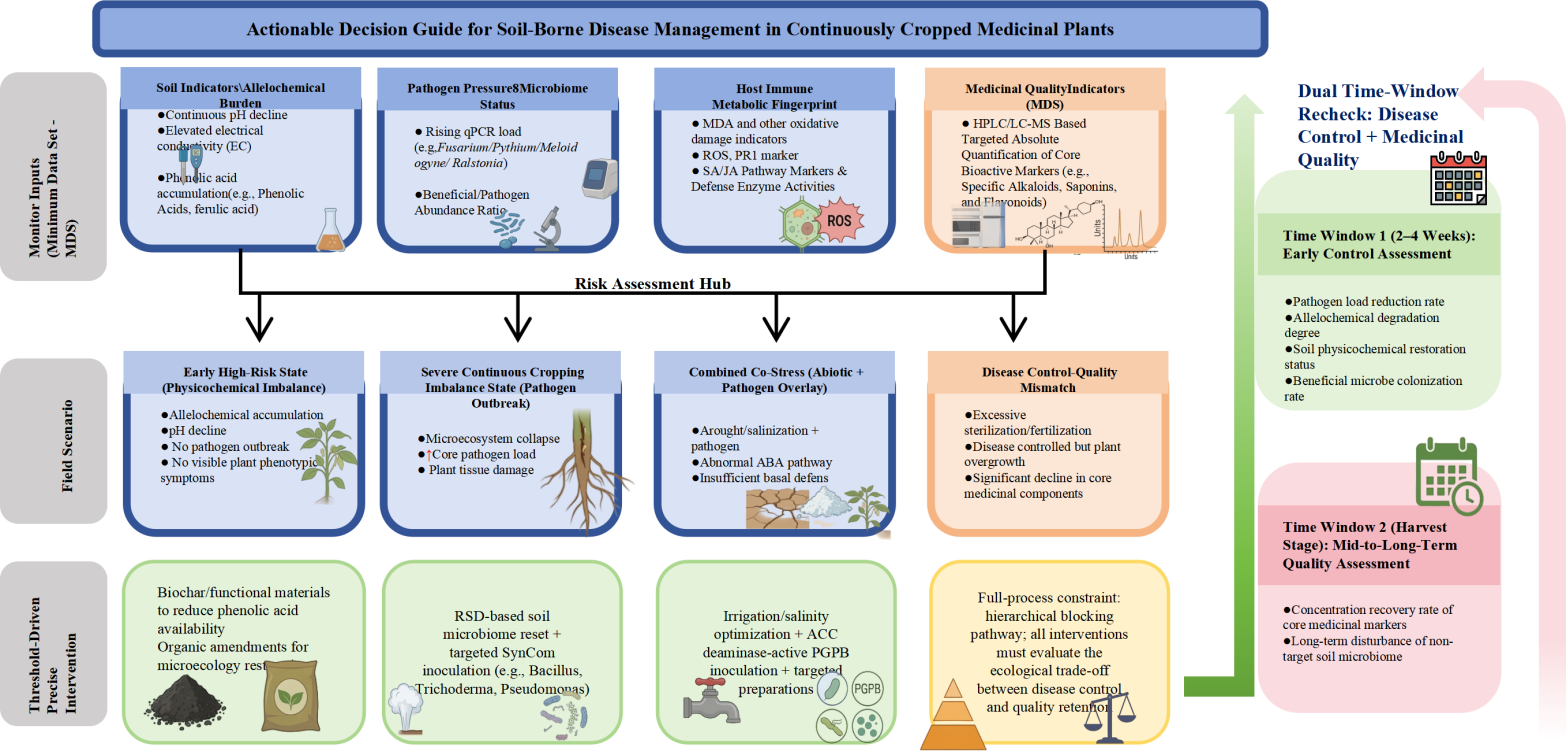
Integrated framework of the actionable decision guide for soil-borne disease control in continuously cropped medicinal plants.

Although multi-omics technologies have improved our understanding of pathogen–environment–host microecological imbalance, translating these findings into practical management strategies is critical for the sustainable control of soil-borne diseases in medicinal plants (**Figure 5**). This strategy focuses on dynamic monitoring and risk management, integrating soil properties, allelochemical load, pathogen pressure (Chen et al., 2023c), microbiome status (Zhou et al., 2023), host immunity (Zhang et al., 2021), and medicinal quality to classify risks and limiting factors, enabling threshold-based precise interventions. Interventions follow the principle of “relieving core stress first, then targeted microecological regulation,” with tailored measures for different scenarios, including soil amendment, RSD, microbial restoration, stress mitigation, and quality protection. Full-process dynamic closed-loop management is adopted to continuously monitor pathogens, soil, host immunity, and quality, unifying disease control, and medicinal quality evaluation to achieve precise disease management and coordinated optimization of yield and quality.

## 8 Discussion

Current studies on soil-borne disease mechanisms in medicinal plants focus mainly on direct host–pathogen interactions involving *Fusarium*, *Rhizoctonia*, and other pathogens, while neglecting the coordinated roles of multi-trophic rhizosphere organisms. No systematic framework exists for identifying pathogen complexes or analyzing dynamic synergistic virulence in complex infections. Moreover, the threshold mechanisms of allelopathic autotoxicity under continuous cropping remain unclear, and quantitative links between root exudate profiles and microbial community shifts are insufficiently defined, warranting future quantitative modeling.

In disease management, the medicinal quality of host plants is often overlooked. Few studies have linked key resistance genes and metabolites to medicinal activity. Integrating disease indices, pathogen dynamics, and bioactive compounds into quantitative models could help identify defense pathways that simultaneously suppress disease and maintain key medicinal components. Supported by multi-omics approaches, research on medicinal plant soil-borne diseases has advanced steadily. Genome editing, combined with high-throughput phenotyping, genomic selection, and rapid breeding, offers a promising route for precision resistance breeding. Modern biotechnologies, such as biochar and microbial agents, can also be integrated with traditional agronomic practices, including rotation and intercropping, to support high-quality variety breeding and sustainable cultivation. Future controlled microbial assembly experiments coupled with multi-omics will help clarify the causal mechanisms of disease development and enable the field application of stable synthetic microbial communities across diverse soils and climatic conditions. Overall, the deep integration of modern biotechnology and conventional agronomy will accelerate the breeding of superior medicinal plant varieties and sustainable cropping systems, promoting industrial upgrading and improving both productivity and sustainability.

Overall, this review integrates recent advances in plant pathology, chemical ecology, and microbiome science and conceptualizes soil-borne disease development in continuously cropped medicinal plants as a coupled process involving pathogen–environment–host interactions. We further linked allelochemical-driven edaphic shifts and rhizosphere dysbiosis with host immunometabolic reprogramming, thereby connecting mechanistic understanding to microbiome-guided management strategies. In the future, controlled community assembly experiments combined with multi-omics approaches can help establish causality and support the translation of robust SynCom formulations for field production across diverse soils and climates. By integrating modern biotechnologies with established agronomic practices, these approaches may accelerate the development of high-quality cultivars and sustainable production systems, ultimately strengthening the medicinal plant sector and improving the economic viability of medicinal herb cultivation.
